# Global trends in incidence, death, burden and risk factors of early-onset cancer from 1990 to 2019

**DOI:** 10.1136/bmjonc-2023-000049

**Published:** 2023-09-05

**Authors:** Jianhui Zhao, Liying Xu, Jing Sun, Mingyang Song, Lijuan Wang, Shuai Yuan, Yingshuang Zhu, Zhengwei Wan, Susanna Larsson, Konstantinos Tsilidis, Malcolm Dunlop, Harry Campbell, Igor Rudan, Peige Song, Evropi Theodoratou, Kefeng Ding, Xue Li

**Affiliations:** 1 Department of Big Data in Health Science, School of Public Health and The Second Affiliated Hospital, Zhejiang University School of Medicine, Hangzhou, Zhejiang, China; 2 Department of Epidemiology, Harvard T.H. Chan School of Public Health, Harvard University, Cambridge, Massachusetts, USA; 3 Department of Nutrition, Harvard T.H. Chan School of Public Health, Boston, Massachusetts, USA; 4 Centre for Global Health, Usher Institute, University of Edinburgh, Edinburgh, UK; 5 Unit of Cardiovascular and Nutritional Epidemiology, Institute of Environmental Medicine, Karolinska Institutet, Stockholm, Sweden; 6 Department of Colorectal Surgery and Oncology, Zhejiang University School of Medicine Second Affiliated Hospital, Hangzhou, Zhejiang, China; 7 Department of Health Management and Institute of Health Management, Sichuan Provincial People's Hospital, University of Electronic Science and Technology of China, Chengdu, China; 8 Unit of Medical Epidemiology, Department of Surgical Sciences, Uppsala University, Uppsala, Sweden; 9 Department of Epidemiology and Biostatistics, School of Public Health, Imperial College London, London, UK; 10 Department of Hygiene and Epidemiology, University of Ioannina School of Medicine, Ioannina, Greece; 11 Colon Cancer Genetics Group, Institute of Genetics and Cancer, University of Edinburgh, Edinburgh, UK; 12 Cancer Research UK Edinburgh Centre, Medical Research Council Institute of Genetics and Cancer, University of Edinburgh, Edinburgh, UK; 13 Centre for Global Health, School of Public Health, Zhejiang University School of Medicine, Hangzhou, China

**Keywords:** Epidemiology, Mortality, Neoplasms

## Abstract

**Objective:**

This study aimed to explore the global burden of early-onset cancer based on the Global Burden of Disease (GBD) 2019 study for 29 cancers worldwid.

**Methods and analysis:**

Incidence, deaths, disability-adjusted life years (DALYs) and risk factors for 29 early-onset cancer groups were obtained from GBD.

**Results:**

Global incidence of early-onset cancer increased by 79.1% and the number of early-onset cancer deaths increased by 27.7% between 1990 and 2019. Early-onset breast, tracheal, bronchus and lung, stomach and colorectal cancers showed the highest mortality and DALYs in 2019. Globally, the incidence rates of early-onset nasopharyngeal and prostate cancer showed the fastest increasing trend, whereas early-onset liver cancer showed the sharpest decrease. Early-onset colorectal cancers had high DALYs within the top five ranking for both men and women. High-middle and middle Sociodemographic Index (SDI) regions had the highest burden of early-onset cancer. The morbidity of early-onset cancer increased with the SDI, and the mortality rate decreased considerably when SDI increased from 0.7 to 1. The projections indicated that the global number of incidence and deaths of early-onset cancer would increase by 31% and 21% in 2030, respectively. Dietary risk factors (diet high in red meat, low in fruits, high in sodium and low in milk, etc), alcohol consumption and tobacco use are the main risk factors underlying early-onset cancers.

**Conclusion:**

Early-onset cancer morbidity continues to increase worldwide with notable variances in mortality and DALYs between areas, countries, sex and cancer types. Encouraging a healthy lifestyle could reduce early-onset cancer disease burden.

**WHAT IS ALREADY KNOWN ON THIS TOPIC**

- Previous studies have suggested that the incidence of cancers of various organs diagnosed in adults<50 years of age has been rising in many parts of the world since the 1990s. The global disease burden and secular trend of early onset cancers, and the variations in different socioeconomic categories, have not been described. The pattern of attributable risk factors for burdensome early onset cancers has not been investigated.

**WHAT THIS STUDY ADDS**

- Since 1990, the incidence and deaths of early onset cancers have substantially increased globally. Early-onset breast, tracheal, bronchus and lung, stomach and colorectal cancers showed the highest mortality and burden in 2019. Countries with a high-middle and middle Sociodemographic Index and individuals aged 40–49 years were particularly affected. Dietary risk factors (diet high in red meat, low in fruits, high in sodium and low in milk, etc), alcohol consumption and tobacco use are the main risk factors underlying early-onset cancers.

**HOW THIS STUDY MIGHT AFFECT RESEARCH, PRACTICE OR POLICY**

- This study suggests that it is necessary to conduct prospective life-course cohort studies to explore the aetiologies of early-onset cancers, and each country should adjust their prevention strategies based on the characteristics of early-onset cancer. Meanwhile, encouraging a healthy lifestyle could reduce early-onset cancer disease burden.

## Introduction

Globally, cancer is a significant cause of morbidity and mortality, resulting in a large disease burden.^1^ According to Global Cancer Statistics 2020, breast cancer with the largest number of 2.3 million new cases accounted for 11.7% of all cancers, followed by lung cancer (11.4%), colorectal cancer (CRC) (10.0 %), while lung cancer was the main cause of cancer death (1.8 million deaths, 18%), followed by CRC (9.4%), liver (8.3%) cancer.^1^ Cancer is generally more prevalent in adults over 50 years, but the incidence of early-onset cancer (<50 years) has increased worldwide.^2^ In comparison to later-onset cancer, the increase of early-onset cancer has significant personal and societal ramifications. Moreover, early-onset cancer and the adverse impacts of some corresponding cancer treatments may result in additional health issues during subsequent life cycle,^3^ which would considerably increase the disease burden associated with early-onset cancers.

Currently, it was reported that the promotion of cancer screening strategies and the exposure to risk factors in early life or young adulthood may increase incidence of early-onset cancer.^4^ For example, increasing the proportion of cervical cancer screening among women aged 21–65 years was a Healthy People 2020 objective for use of cancer screening tests in the USA,^5^ and the American Cancer Society (ACS) has recommended initiating CRC screening at age 45 years instead of 50 years.^6^ For breast cancer, in countries with favourable healthcare conditions, it is recommended that women between the ages of 40 and 49 undergo a screening test for breast cancer every 1–2 years.^7–11^ Furthermore, changes in diet, lifestyle and environment since the turn of the 20th century, resulting in increased rates of obesity, physical inactivity, westernised diets and environmental pollution, may have affected the incidence of early-onset cancer.^12^ Additionally, alcohol, smoking and detrimental pregnancy exposures may have also affected the incidence of early-onset cancer.^2 13^

The majority of previous studies focused on regional and national variations in the incidence and death of all-age cancer,^14^ and only a small number of studies examined the worldwide epidemiology and disease burden of early-onset cancer. A systematic examination on the global epidemiology of early-onset cancer can aid in the efficient implementation of prevention, early detection, diagnosis and treatment initiatives. Thus, we conducted this study to describe the global burden of early-onset cancer based on the Global Burden of Disease (GBD) 2019 study for 29 cancers in 204 countries and regions with the aim of shedding light on early-onset global cancer prevention and control.

## Material and methods

### Data source, definition of early-onset cancer and risk factors

We obtained the data from the GBD 2019 database (http://ghdx.healthdata.org/gbd-2019). The incidence, deaths, disability-adjusted life years (DALYs) and risk factor proportion were extracted directly from GBD 2019. All rates are reported per 100k population. A total of 29 early-onset cancers were ascertained from administrative data according to International Classification of Disease 9th revision (ICD-9) and 10th revision (ICD-10) codes, the ICD codes of 29 cancers are shown in online supplemental table S1. Early-onset cancer was defined as cancer cases diagnosed from 14 to 49 years.^15^ In total, 204 countries were divided into five-level regions based on the Sociodemographic Index (SDI). The Human Development Index (HDI) data were obtained at the national level from the World Bank. Definitions of risk factors and method for calculating the proportions of their attribution in GBD 2019 were detailedly described in online supplemental methods.

10.1136/bmjonc-2023-000049.supp1 Supplementary data

### Statistical analysis

The incidence rate, death rate and estimated annual percentage change (EAPC) were used to quantify the epidemic trends of 29 early-onset cancers. The EAPC formulas were as follows:

y=α+βx+ε

EAPC=100×(exp(β)−1)

The age-standardised rate (ASR)/100k population, including age-standardised incidence rate (ASIR) and age-standardised death rate (ASDR), was estimated with the following formula:

$$ASR=\frac{\sum_{i=1}^{A}a_{i}W_{i}}{\sum_{i=1}^{A}W_{i}}\times 100k$$

The selected reference standard population was presented in online supplemental table S2. We explored the associations between EAPCs and HDI in 2019 using Spearman correlation analysis. Additionally, the Bayesian age-period-cohort (BAPC) model integrating nested Laplace approximations was used to project the morbidity and mortality of the disease burden attributable to early-onset cancer from 2020 to 2030.^16^ The BAPC model is widely used in analysing and projecting age-stratified cancer incidence and mortality rates, particularly considering the significant demographic changes taking place. The primary advantage of developing the BAPC package is to create efficient Markov chain Monte Carlo-free software specifically designed for routine utilisation in epidemiological applications. This package simplifies the implementation of the BAPC model, enabling the generation of well-calibrated probabilistic forecasts with reasonably narrow ranges of uncertainty. The formula was as follow:

$$R_{ijk}=\mu +\alpha_{i}Age+\beta_{j}Period+\gamma_{k}$$

To avoid over dispersion, an independent random effect, $z_{ij}∼\left(0,k_{z}^{-1}\right)$ , was added into the model:

$$R_{ijk}=\mu +\alpha_{i}Age+\beta_{j+t}Period+\gamma_{k+t}Cohort+z_{ij+t}$$

Data analysis and graphics were conducted using R V.4.2.1 (Lucent Technologies, Jasmine Mountain, USA). P value<0.05 was considered to be statistically significant. All parameters were detailedly described in the online supplemental methods.

### Patient and public involvement statement

The GBD Study is a global collaborative scientific effort involving more than 7500 people from about 150 countries. We did not consider involving patients when designing the study and no patients were involved in setting the specific research question, collecting and analysing the data, interpreting the results, or writing up the manuscript. The research findings will be disseminated to the wider community by press releases, social media platforms such as WeChat, presentations at international fora, reports to relevant government agencies and academic societies.

## Results

### Trends in incidence, death and DALYs of 29 early-onset cancers from 1990 to 2019

**Figure 1:**
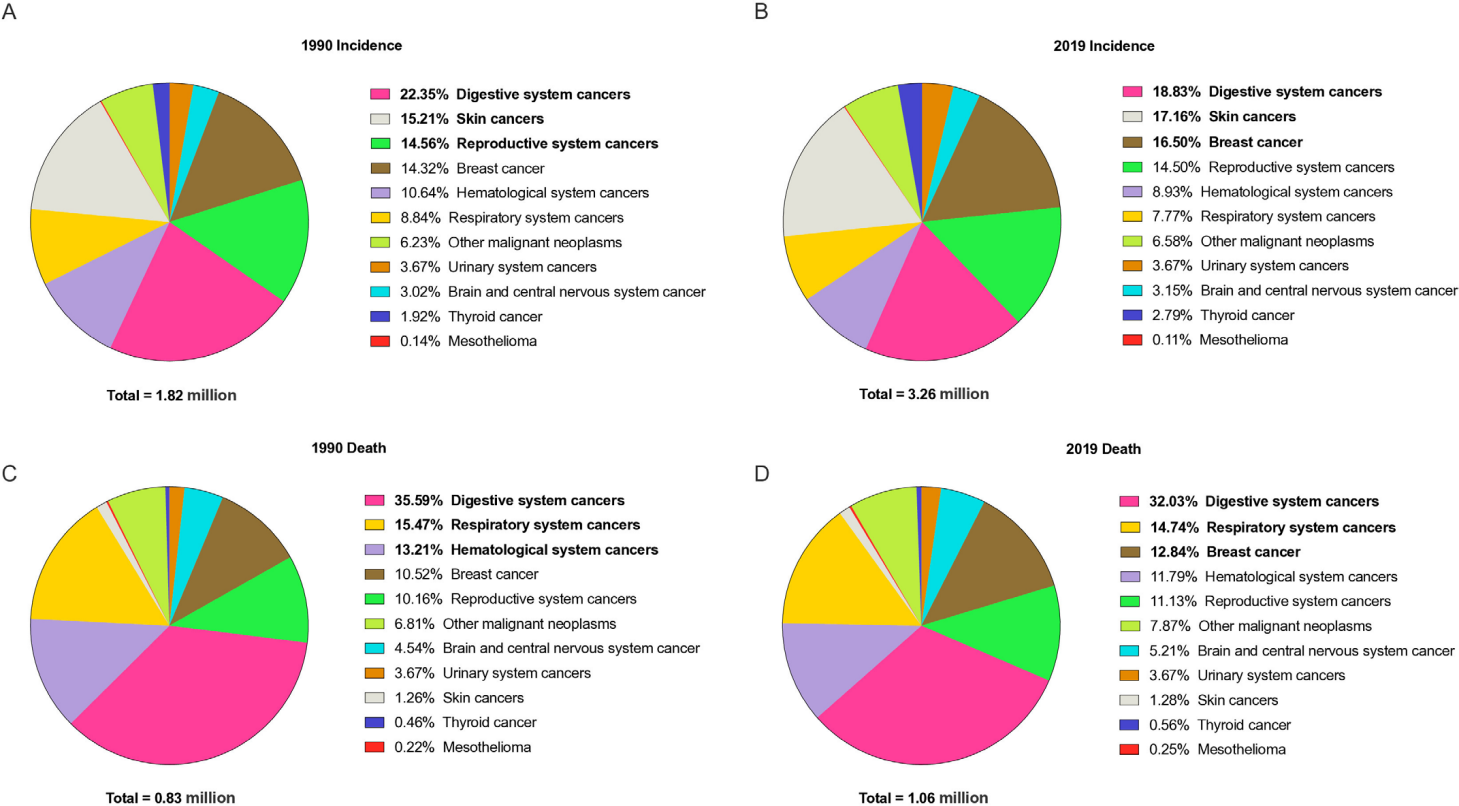
Distribution of cases and deaths for the early-onset cancers of different systems in 1990 and 2019. The early-onset cancer types in bold are the top three. Respiratory system cancers include larynx cancer, nasopharynx cancer, other pharynx cancer and tracheal, bronchus, and lung cancer; digestive system cancers include colon and rectum cancer, oesophageal cancer, gallbladder and biliary tract cancer, lip and oral cavity cancer, liver cancer, pancreatic cancer and stomach cancer; haematological system cancers include Hodgkin’s lymphoma, leukaemia, multiple myeloma and non-Hodgkin’s lymphoma; reproductive system cancers include cervical cancer, ovarian cancer, testicular cancer and uterine cancer; urinary system cancer include bladder cancer, kidney cancer and prostate cancer; skin cancers include malignant skin melanoma and non-melanoma skin cancer.

In 2019, the incidence number of early-onset cancer was 3.26 million, a 79.1% increase from 1990 (figure 1A,B). Among them, the early-onset breast cancer had the highest incidence (13.7, 95% uncertainty interval (UI): 12.5 to 15 per 100k) and mortality (3.5, 95% CI: 3.2 to 3.8 per 100k) rates (online supplemental tables S3 and S4). Globally, the morbidity of early-onset nasopharyngeal cancer (EAPC=2.28%, 95% CI: 2.1% to 2.47%) and prostate cancer (EAPC=2.23%, 95% CI: 1.97% to 2.49%) showed the fastest increasing trends, whereas early-onset liver cancer (EAPC=−2.88%, 95% CI: −3.46% to –2.3%) showed the sharpest decline (online supplemental table S3). Besides, the number of early-onset cancer deaths in 2019 was 1.06 million, which was an increase of 27.7% from 1990 (figure 1C,D). The top four early-onset cancers with the highest mortality and DALYs rates were early-onset breast, tracheal, bronchus and lung (TBL), stomach and CRC cancers (online supplemental tables S4 and S5). The mortality of early-onset kidney cancer (EAPC=0.81%, 95% CI: 0.70% to 0.92%) and ovarian cancer (EAPC=0.59%, 95% CI: 0.49% to 0.69%) showed the fastest increasing trends, whereas early-onset liver cancer (EAPC=−3.39%, 95% CI: −4.00% to –2.77%) showed the sharpest decline (online supplemental table S4). In 2019, early-onset breast cancer had the highest ASIR in regions with high SDI, while early-onset TBL cancer had the highest ASIR in high-middle SDI regions (online supplemental table S6). Early-onset CRC had the highest ASIR in high SDI regions, and stomach cancer had the highest ASIR in high-middle SDI regions in 2019. On the other hand, the highest ASDR for early-onset breast cancer were observed in regions with low and low-middle SDI in 2019. Early-onset TBL cancer had the highest ASDR in high-middle SDI regions in 2019, while early-onset CRC and stomach cancer had the highest ASDR in high-middle and low-middle SDI regions, respectively. As depicted in online supplemental figure S1, the morbidities, mortality and DALYs rates of early-onset breast cancer and CRC increased simultaneously from 1990 to 2019.

### Early-onset cancer burden differed by sex

**Figure 2:**
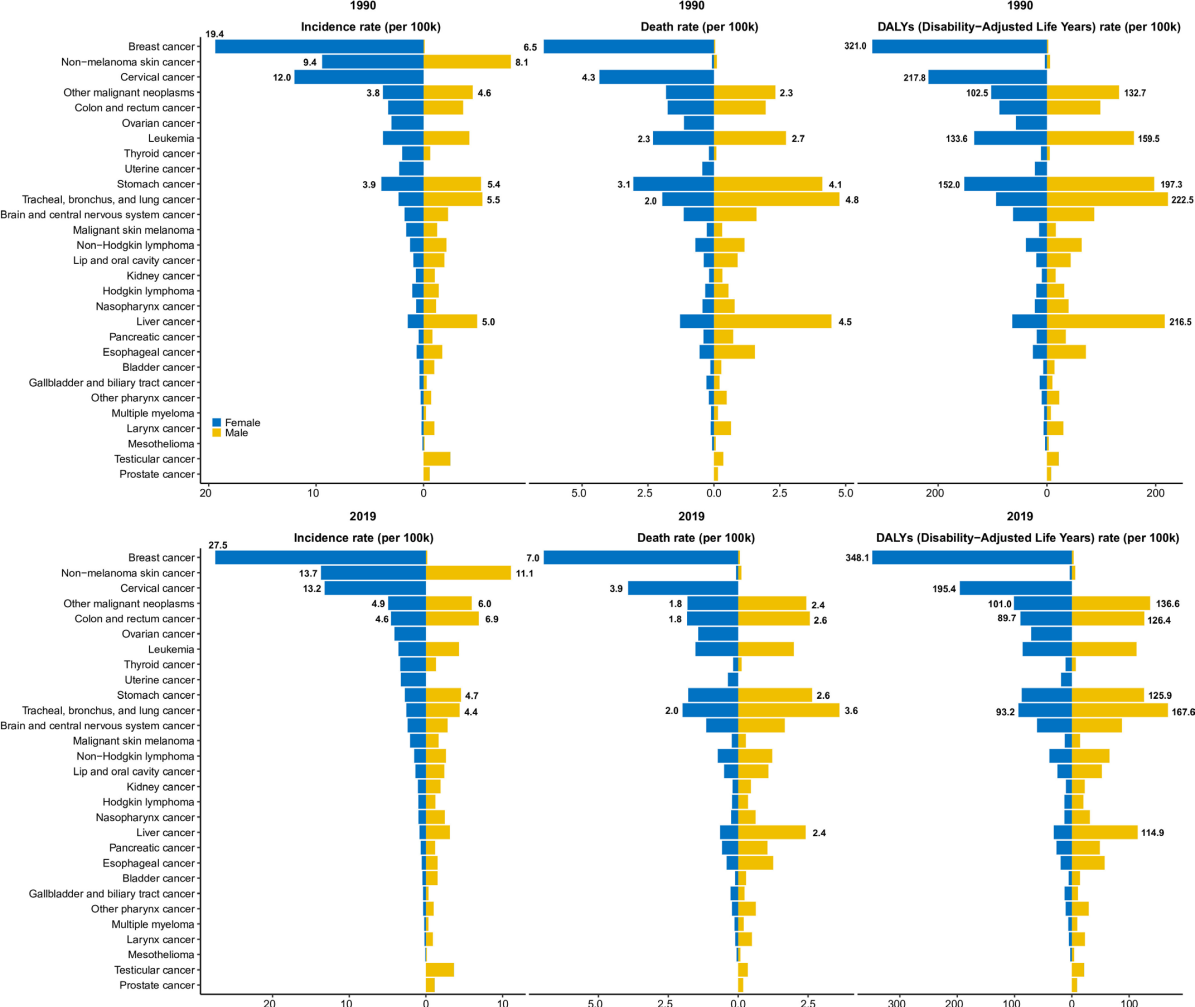
The global incidence, death and DALYs rates of 29 specified early-onset cancers in 1990 and 2019 by sex. DALYs, disability-adjusted life years.

In 2019, the early-onset cancers with the greatest disease burden in women and men were breast cancer (348.1, 95% UI: 316.7 to 378.7 per 100k) and TBL cancer (167.6, 95% UI: 149.9 to 186.5 per 100k), respectively (figure 2). The men/women ratios of morbidity, mortality and DALYs below 1 meant higher burden in women, while the men/women ratios above 1 meant higher burden in men. For morbidity, the men/women ratios in five SDI regions were consistently below 1 from 1990 to 2019 and showed a downward trend (online supplemental figure S2A), which suggested that the general morbidity of early-onset cancer in men was lower than women from 1990 to 2019. And men/women ratios of mortalities and DALYs were close to 1 in high, high-middle and middle SDI regions, 2019, while were far less than 1 in low-middle and low SDI regions (online supplemental figure S2B and S2C). The results above indicated that, in low-middle and low SDI regions, early-onset cancer had a significantly higher impact on women than on men in terms of both mortality and disease burden.

### Early-onset cancer burden in different world regions

In 2019, the highest ASIR of early-onset cancer were in high-income North America (273.2 per 100k), while the lowest ASIR were in Western Sub-Saharan Africa (37.4 per 100k) (online supplemental table S7). The regions with the highest ASDR were Oceania (39.1 per 100k), Eastern Europe (33.7 per 100k) and Central Asia (31.8 per 100k), whereas the lowest were in high-income Asia Pacific (16.3 per 100k) (online supplemental table S7). Of note, East Asia’s incident and death numbers of early-onset cancer were 814 749 and 268 709, respectively, ranking first among all regions. The greatest age-standardised DALYs rates were in Oceania (1952.6 per 100k), while the lowest were in high-income Asia Pacific (840.6 per 100k) in 2019 (online supplemental table S7).

### ASIR, ASDR and age-standardised DALYs in 2019, and relative change in incident, death and DALYs cases of early-onset cancers from 1990 to 2019

**Figure 3:**
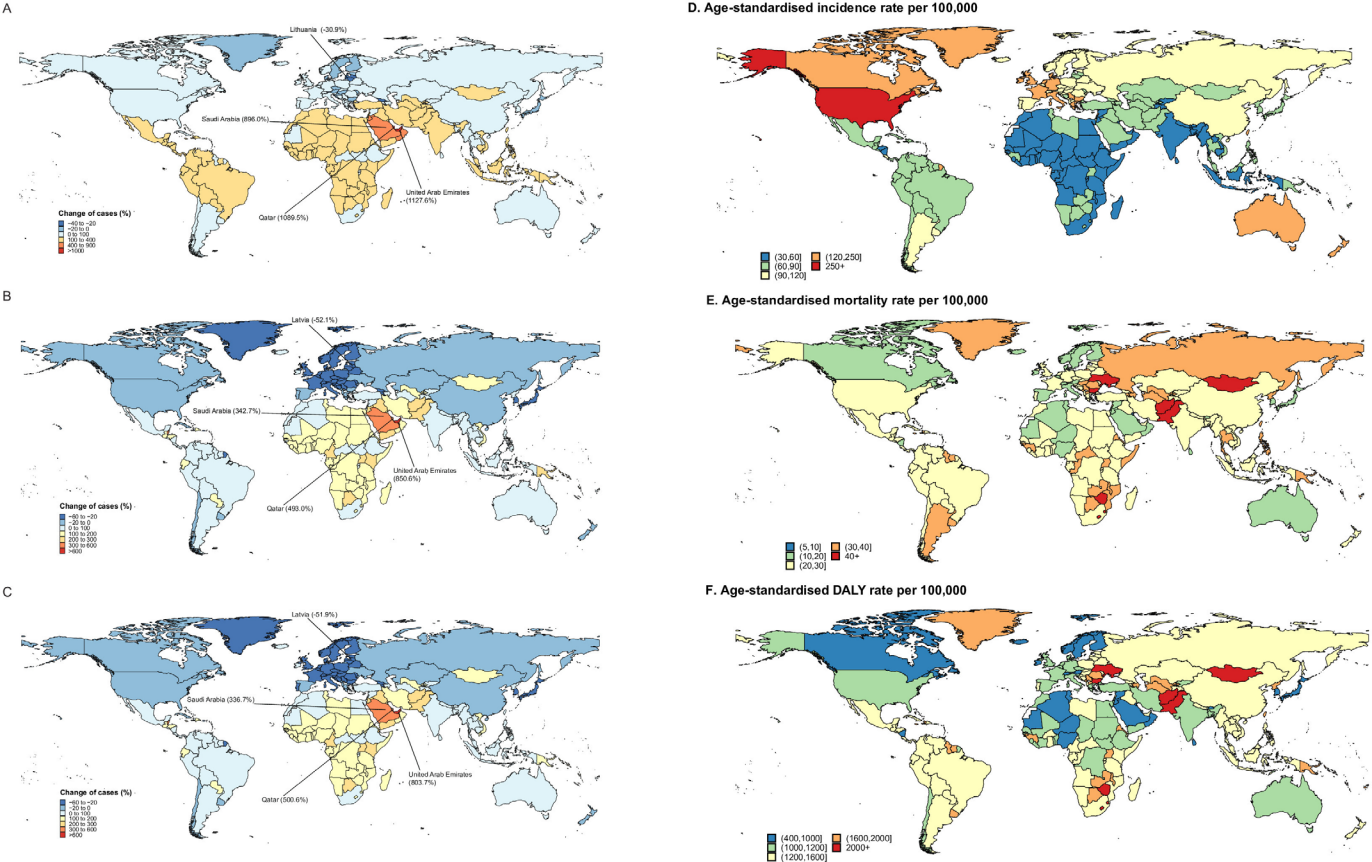
Among 204 countries and territories, the relative change of incident (A), death (B) and DALYs (C) cases of early-onset cancers from 1990 to 2019, and ASIR (D), ASDR (E), age-standardised DALYs rate (F) in 2019. ASIR, age-standardised incidence rate; ASDR, age-standardised death rate; DALYs, disability-adjusted life years.

Globally, the incident and death cases of early-onset cancers increased by 79.0% and 28.5% in 2019, respectively (figure 1). The United Arab Emirates (1127.6%), Qatar (1089.5%) and Saudi Arabia (896.0%) exhibited the sharpest increases in the number of incident cases from 1990 to 2019, while Lithuania decreased by 30.9%, followed by Georgia (−30.0%) and Latvia (−29.0%) (figure 3A). Besides, the most pronounced change in the number of death and DALYs cases was observed in the United Arab Emirates (850.6% and 803.7%), while Latvia (−52.1% and −51.9%) experienced the greatest decline (figure 3B,C). In 2019, the highest ASIR and ASDR of early-onset cancer were in the USA (282.1 per 100k) and Solomon Islands (82.9 per 100k) and the lowest ASIR and ASDR were in Niger (31.0 per 100k) and Kuwait (9.5 per 100k) (figure 3D,E). Moreover, 20 countries have an age-standardised DALYs of more than 2000 per 100k (figure 3F).

Furthermore, the global and regional proportions of death number among 29 early-onset cancers in 2019 are described in online supplemental figure S3.

### The trend of ASIR, ASDR and DALY rate of all early-onset cancers in regions and countries with different SDI levels

The overall trend indicated that the morbidity of all early-onset cancers gradually increased with the SDI (online supplemental figure S4A). When SDI was below 0.7, the ASDR and DALYs of all early-onset cancers increased with the SDI value and the ASDR and DALYs then decreased considerably when SDI kept increasing after 0.7 (online supplemental figure S4B and S4C). At the national level, the association between SDI and the ASIR of all early-onset cancers was similar to the regional level (online supplemental figure S5).

### Composition and trend of early-onset cancer incident cases in the digestive and respiratory system

In 2019, after breast cancer, the digestive and respiratory systems of early-onset cancer were mainly responsible for the deaths (figure 1). In digestive system globally, early-onset stomach cancer (30.9%) was the largest proportion of early cancer in 1990, follow by early-onset CRC (23.3%) and liver cancer (21.6%); however, in 2019, early-onset CRC (36.8%) has surpassed stomach cancer as the most numerous form of early cancer (online supplemental figure S6A). In addition, although TBL cancer has been the most important early-onset cancer of the respiratory system from 1990 to 2019, early-onset nasopharyngeal cancer increased from 15.7% in 1990 to 26.8% in 2019, particularly in high-middle SDI region (online supplemental figure S6B).

### The association between EAPC and HDI in the most burdensome early-onset cancers

As shown in online supplemental figure S7A, significant association was observed among EAPC_morbidity_, EAPC_mortality_ and HDI (in 2019) for early-onset breast cancer, TBL cancer, CRC and stomach cancer, respectively. For EAPC_morbidity_ and EAPC_mortality_ in these early-onset cancers, a significant positive association was found between EAPC and HDI when the HDI was limited to below 0.6 or 0.7; a significant negative relation was detected between EAPC and HDI when the HDI was greater than 0.6 or 0.7 (online supplemental figure S7).

### Risk factors for early-onset breast cancer, TBL cancer, CRC and stomach cancer

**Figure 4:**
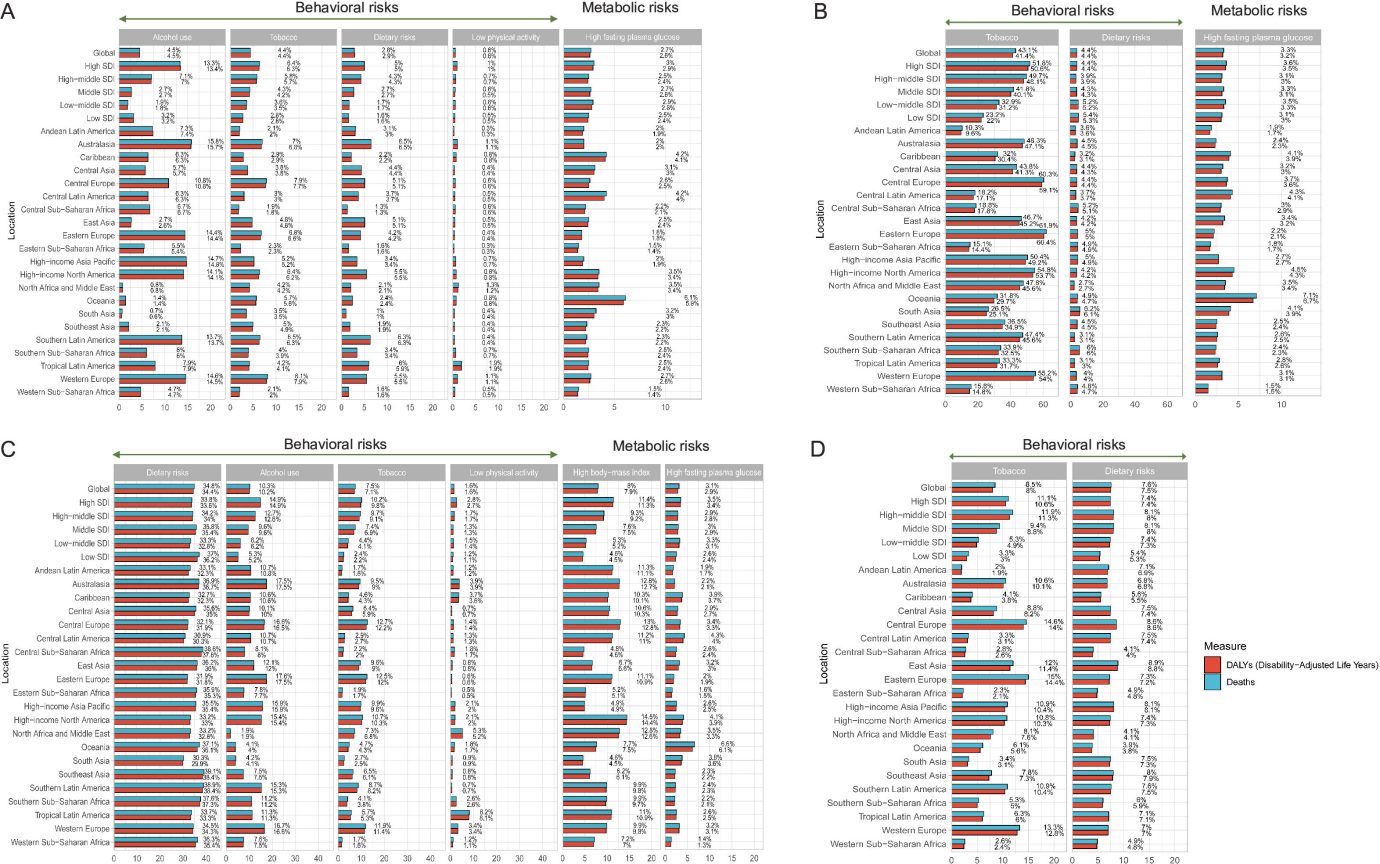
The risk factors of early-onset breast cancer (A), TBL cancer (B), CRC (C) and stomach cancer (D) worldwide, in 21 GBD and 5 SDI regions, 2019. CRC, colorectal cancer; DALYs, disability-adjusted life years; GBD, Global Burden of Disease; SDI, Sociodemographic Index; TBL, tracheal, bronchus and lung.

We explored the behaviour and metabolic risk factors for death and DALYs of early-onset cancers with the highest disease burden in 2019 (figure 4). Globally, the leading risk factors for early-onset breast cancer DALYs were alcohol use (4.5%, 95% UI: 3.7% to 5.5%), tobacco smoking (4.4%, 95% UI: 1.9% to 6.6%), diet high in red meat (2.9%, 95% UI: 1.4% to 3.8%), physical inactivity (0.6%, 95% UI: 0.3% to 1.2%) and high fasting plasma glucose (2.6%, 95% UI: 0.5% to 6.4%) (figure 4A and online supplemental figure S8A). For early-onset TBL cancer DALYs, tobacco smoking (41.4%, 95% UI: 37.7% to 45.5%) was the most important risk factor, followed by diet low in fruits (4.4%) and high fasting plasma glucose (3.2%) (figure 4B and online supplemental figure S8B). Six risk factors were identified for early-onset CRC, including dietary risks, alcohol use, tobacco smoking, low physical activity, high body mass index (BMI) and high fasting plasma glucose. Dietary risks for DALYs globally reached 34.4%, mainly consisting of a diet low in milk (16.5%), low in whole grains (15.2%) and low in calcium (14.3%) (figure 4C and online supplemental figure S8C). The percentage trends of diet low in milk, low in calcium and low in whole grains-attributable DALYs and deaths in early-onset CRC from 1990 to 2019 are shown in online supplemental figure S9. Tobacco smoking (8.0%) and a diet high in sodium (7.5%) were the risk factors for early-onset stomach cancer globally (figure 4D and online supplemental figure S8D). The ranking change of DALYs risk factors for early-onset cancer from 1990 to 2019 among women and men is presented in online supplemental figure S10. The results indicated that the ranking of risk factors in 2019 for early-onset breast cancer (online supplemental figure S10A), cervical cancer (online supplemental figure S10B) and stomach cancer (online supplemental figure S10C) in women has not altered, compared with 1990. For early-onset CRC in women, the ranking of alcohol use, tobacco, a diet low in fibre and low physical activity in 2019 downgraded compared with 1990, while a diet high in red meat, high BMI and high fasting plasma glucose upgraded (online supplemental figure S10D). The ranking of risk factors for early-onset TBL cancer (online supplemental figure S10E) and stomach cancer in men (online supplemental figure S10G) did not change. In 2019, the ranking of dietary factors associated with early-onset CRC in men underwent some changes when compared with the rankings in 1990. Specifically, a diet low in calcium and fibre was downgraded, while a diet low in milk and whole grains, along with high BMI and high fasting plasma glucose, were upgraded (online supplemental figure S10F). Meanwhile, for early-onset liver cancer in men, the rankings of tobacco and alcohol use had been interchanged in 2019 (online supplemental figure S10H).

### Prediction of the incidence and death of early-onset cancers from 2020-2030 worldwide

**Figure 5:**
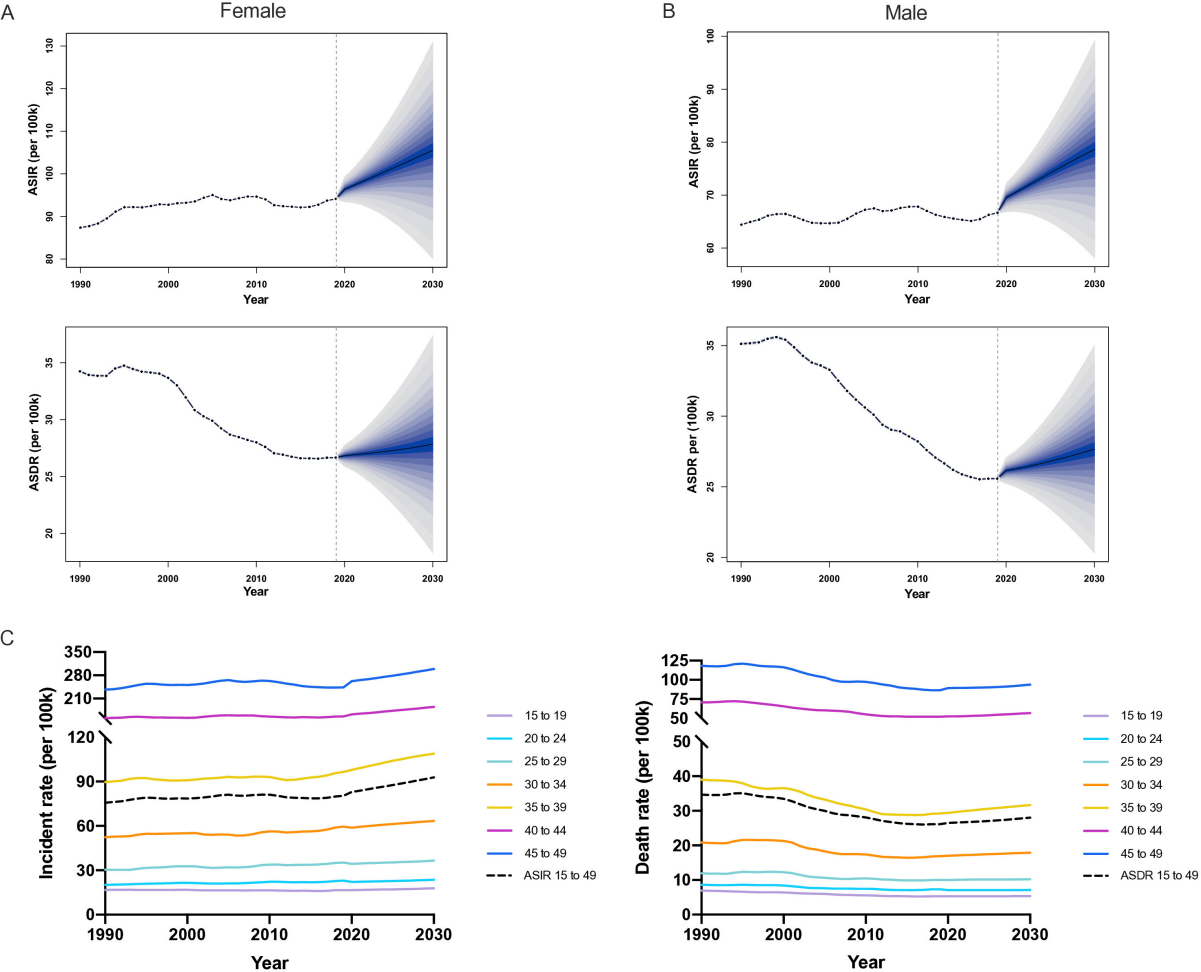
Trends of ASIR and ASDR in the early-onset cancer in female (A), male (B) and both sex (C): observed (1990–2019) and predicted rates (2020–2030). The blue region in (A) and (B) shows the upper and lower limits of the 95% uncertainty intervals (95% UI). ASIR, age-standardised incidence rate; ASDR, age-standardised death rate.

The ASIR of early-onset cancer in women were higher than in men in 2020–2030, the increasing trend in ASIR and ASDR of early-onset cancer for men were similar to women (figure 5A,B and online supplemental figure S11). Furthermore, the 40–44 and 45–49 age groups were substantially the major population of early-onset cancer morbidity and mortality from 1990 to 2030 (figure 5C). The ASIR of early-onset cancer would continue to increase globally from 2020 to 2030. The ASDR showed a slight increase compared with 2019 (figure 5C), especially in men.

## Discussion

The study systematically evaluated the global burden of 29 cancers with early onset in 2019. The analysis of incidence, death and DALYs by location, country and sex revealed that the spectrum of early-onset cancer varied significantly among the regions and nations across the world. Although the global early-onset cancer incidence surpassed 3.26 million in 2019, a 79.1% increase of the incidence in 1990, the mortality number of early-onset cancer only increased by 27.7%. Notably, the prediction model indicated that the age brackets of 40–44 and 45–49 will represent a significant proportion of the population affected by early-onset cancer morbidity and mortality in the next 10 years.

Significant regional variations in the early-onset cancer spectrum can be ascribed to the local environment, lifestyle and level of available medical treatment. For example, in high-income North America, Australasia and Western Europe with high degree of development, the ASIRs in 2019 were higher than 125 per 100k, while the lowest were in Western Sub-Saharan Africa and Central Sub-Saharan Africa (<50 per 100k), which were similar to the ASIR of all-age cancer.^14^ At present, cancer control efforts, preventive measures and strategies in Africa are inadequate, and the majority of Africa countries have limited cancer registries, and their cancer reporting systems are poorly organised.^17^ Besides, most countries in Africa do not have well established health insurance systems to cover the cost of cancer screening.^18^ The afore-mentioned factors could be contributing factors to the Africa’s low ASIRs for early-onset cancer. On the whole, the more developed the country and region, the higher the incidence of early-onset cancer. The rising incidence of early-onset cancers may partially attribute to increasing uptake of screening and early detection in developed regions and countries^2^; however, only a small number of countries and certain types of cancer (including cervical cancer, breast cancer and CRC^19^) have implemented a screening strategy for individuals with cancer who are under the age of 50. Beyond this, western diet risk and lifestyle risk factors promoted the incidence rate of early-onset cancers.^1^ However, as SDI values increased, there was a sharp decrease in ASDR for early-onset cancer in Western Europe, high-income Asia Pacific and high-income North America regions. Furthermore, the projections indicated that the global morbidity and ASIR from early-onset cancer would increase over the next 10 years, while there would be a slight increase in mortality and ASDR compared to 2019 due to changing demographics over the following 10 years. Our results suggested that the incidences of early-onset nasopharynx cancer and prostate cancer displayed the most rapid upward trends in morbidity from 1990 to 2019. Despite the regional clustering and ethnic susceptibility of nasopharynx cancer, its underlying causes are currently unknown. While genetic factors, Epstein-Barr virus (EBV) infection and environmental factors are thought to be significant contributors to the development of nasopharynx cancer, more research is required to establish exact aetiological roles of early-onset nasopharynx cancer.^20^ Prostate-specific antigen (PSA) screening, which began in developed countries in the 1990s, contributed to the incidence of early-onset prostate cancer.^20^ However, it would be incorrect to solely attribute the entire increase in early-onset prostate cancer since 1986 to PSA screening. Besides advancements in screenings and diagnostics, other possible reasons for variations in health outcomes could be differences in age demographics and the presence of genetic and lifestyle risk factors.^20^

Among 29 early-onset cancers, breast cancer had the highest morbidity, mortality and DALYs. In 1990, North America regions with high-income levels had the highest rate of early-onset breast cancer (30.6 per 100k), but by 2019, the incidence of early-onset breast cancer (23.1 per 100k) had decreased, although it still remained in third place. It may benefit from the application of early screening programmes of breast cancer in North America regions. In contrast, over the same time period, Asia regions experienced a significant increase in the incidence of early-onset breast cancer, rising from 4.9 to 13.1 per 100k in 1990 to 8.7–15.6 per 100k in 2019. The growing prevalence of a westernised lifestyle could be among the factors contributing to the upward trend observed in Asian countries.^21 22^ Recently, a case–control study of Asian American women (diagnosed at age≤55 years) from the San Francisco Bay Area found that breast cancer risk was marginally increased among foreign-born women (OR=1.40) and twofold among foreign-born Chinese women.^23^ Thus, the factors (including genetic susceptibility) driving the increasing burden of breast cancer in women of Asian are still unclear. Furthermore, the death of early-onset breast cancer accounted for 32% and 20% among all early-onset cancers in the Oceania and Southeast Asia, respectively. The extensive application and promotion of mammography screening worldwide from 2005 to 2015^24^ has led to an earlier age of breast cancer screening and higher rates of early-onset breast cancer detection. The most typical country is the USA,^25^ which began to introduce and promote mammography screening from 1980s. The ACS suggests that women be given the chance to commence yearly screening between the ages of 40–44, and to undergo routine screening mammography from the age of 45 onwards.^20^ More importantly, it is noteworthy that the incidence of early-onset breast cancer also increased in some countries without the introduction of routine screening,^26^ suggesting that the change of reproductive factors (younger age at menarche, oral contraceptive use, nulliparity, older age at first birth and never breast feeding), physical indicators (higher BMI) and behaviour factors (physical inactivity and alcohol consumption) during recent decades may have contributed to the increasing incidence of early-onset breast cancer.^2^ Globally, we found that alcohol use and tobacco were always the leading risk factors for early-onset breast cancer DALYs during 1990–2019. Several previous studies also found that both tobacco use and alcohol consumption increase the risk of developing breast cancer, with tobacco use specifically linked to premenopausal breast cancer^27^ and alcohol consumption linked to increased risk regardless of menopausal status.^28^ The above evidences highlights that limiting and quitting alcohol and tobacco may serve as a promising strategy to reduce the growing burden of early-onset breast cancer.

Early-onset TBL cancer had the highest burden in men and secondary cause of death for the overall population. Generally, the incidence of early-onset TBL cancer dropped during 1990–2019, which benefited from tobacco control in recent decades.^29^ In 1990, the regions of Central Europe, Eastern Europe, Central Asia, high-income North America and East Asia had the highest ASIR of early-onset TBL cancer among all regions. However, ASIR of early-onset TBL cancer in these regions decreased by 2019. Notedly, lung adenocarcinoma in East Asia, especially among those who have never smoked, tends to have an early onset, which sets it apart from cases observed in other regions.^30^ The differences in incidence rates may be due to various risk factors, such as genetics and exposure to environmental pollution.^31 32^ Thus, by examining the molecular characteristics and defining the hallmarks of tumour progression in early-onset TBL cancer, precision medicine and prevention may be a viable approach for managing non-smoking early-onset TBL cancer in East Asia. Globally, the morbidity and mortality of early-onset TBL cancer in men was 1.7 and 1.8 times higher than that of women, respectively, which was mainly attributed to the higher tobacco consumption in men.^33^ Notably, between 1990 and 2019, smoking continued to be the most significant risk factor for lung cancer among men. Currently, the definition of high-risk or moderate-risk individuals in National Comprehensive Cancer Network (NCCN) Clinical Practice Guidelines in Oncology for lung cancer screening was restricted to those aged 50 years or older.^34^ However, it is still to be assessed if lung cancer screening is necessary for populations with a history of long-term and high-dose smoking who are younger than 50 years old. TBL cancer is similarly caused by passive exposure to tobacco smoking, environmental pollution and indoor lampblack pollution,^35^ particularly in women. And outdoor air pollution may be emerging as an important risk factor for early-onset TBL cancer.^36 37^ In addition to tobacco, we identified two risk factors for early-onset TBL cancer: high fasting plasma glucose and a diet low in fruits. Recently, a meta-analysis of prospective cohort studies demonstrated high glycaemic index diet increased risks of lung cancer.^38^ Therefore, it is necessary to implement a planned programme of measures, including preventing indoor and outdoor air pollution, promoting balanced diet and blood glucose control for diabetic, to further reduce the burden of early-onset TBL cancer.

Early-onset CRC also had high DALYs for both sexes and was the most common form of digestive system early-onset cancer in 2019 globally, accounting for 36.8%. It was reported that greater proportions of patients younger than 50 years were diagnosed with advanced-stage tumours than older patients, thus promoting diagnosis of early-onset CRC patients and identification of potential risk factor were important to improving prevention and therapy of early-onset CRC.^39^ In 1990, Australasia had the highest occurrence of early-onset CRC. By 2019, this incidence had further increased. However, it is important to highlight that in East Asia, the ASIR of early-onset CRC rose from 4.2 per 100k in 1990 to 10.0 per 100k in 2019, making it the top-ranked region. Besides, the results of risk factors analysis indicated that a diet low in milk, low in whole grains and low in calcium were the top risk factors for early-onset CRC in both women and men. Although diet low in calcium were the top risk factors for early-onset CRC in 2019, its risk proportion showed a general downward trend from 1990 to 2019 across all SDI regions, which may benefit from calcium fortification programme since 1990.^40^ Interestingly, as one source of calcium intake, an obvious downward trend of risk proportion of diet low in milk of early-onset CRC from 1990 to 2019 was only observed in high SDI region, but an upward or flat trend in other SDI regions. For a diet low in whole grains, the risk proportion in high SDI region has been on an upward trend from 1990 to 2019. The above results suggest that calcium and milk fortification should be taken into reducing the risk of early-onset CRC in the population, especially in non-high SDI regions. Furthermore, diet in whole grains should be promoted, especially in high SDI regions. Except for dietary risk factors, alcohol use, high BMI, tobacco consumption, high fasting plasma glucose and low physical activity contributed to early-onset CRC. Of these risk factors, high BMI, particularly obesity, has been identified as a strong risk factor for early-onset CRC. The increasing prevalence of obesity in younger generations has led to a substantial increase in early-onset CRC cases.^41^ According to research, obesity is associated with an OR of 1.4 for early-onset CRC.^42^ Besides, individuals with high fasting plasma glucose and diabetes have a higher risk of developing early-onset CRC, as demonstrated by previous studies,^43^ and it was recommended to conduct CRC screening earlier for those with diabetes than for the general population.^44^ Taken together, in addition to focusing on traditional lifestyle risk factors, dietary modifications will have a positive impact on lowering the incidence burden of early-onset CRC.

The ASIR of early-onset stomach cancer in 2019 was highest in East Asia, high-income Asia Pacific, and Eastern Europe, whereas Oceania had the highest ASDR. Generally, stomach non-cardia cancer was common in Eastern Asia and Eastern Europe where the prevalence of *Helicobacter pylori* infection is quite high.^45^ Overall, the morbidity and mortality of early-onset stomach cancer in the most regions showed a downward trend from 1990 to 2019, which suggests that the prevention and treatment of stomach cancer has achieved a remarkable success in recent decades. Undoubtedly, the decrease in mortality of early-onset stomach cancer mainly attributed to the control of risk factors, screening, and treatment methods. For instance, the prevalence of *H. pylori* infection, associated with early-onset stomach cancer, has declined in the USA, most European countries and several East Asian countries.^46^ More importantly, surgical resection combined with neoadjuvant/perioperative chemotherapy is a highly effective treatment for stomach cancer in early stage, which gives a substantial support to the prevention and treatment of early stomach cancer. Additionally, our result indicated that tobacco and a diet high in sodium were the main risk factors for early-onset stomach cancer. Therefore, the morbidity of early-onset stomach cancer might benefit from the decrease in salt intake and tobacco control.

As the SDI increased, there was a rise in the ASIR of early-onset cancer; yet in regions with a high SDI (>0.7), there was a reduction in both early-onset cancer ASDR and DALYs rate as the SDI increased. The high of ASDR and DALYs rate concentrated on middle and middle-high SDI regions. Despite the fact that the ASIR remained elevated in high SDI regions, advancements in medical technology and treatments had substantially alleviated the mortality and overall impact of early-onset cancer. Conversely, it appeared that the middle SDI region was afflicted by the issue of early-onset cancer with high deaths and DALYs. Meanwhile, our findings indicated that the relationship between the HDI and the EAPC of incidence and death rates for early-onset breast cancer, TBL cancer, CRC and stomach cancer follows an inverted U-shaped curve, and the highest EAPC was observed in regions with low-middle and middle HDI, ranging from 0.5 to 0.7. Therefore, it could be concluded that in countries with a low-middle and middle HDI, burdensome early-onset cancers were displaying the most rapid increase in incidence, mortality and DALYs rates. As a result, enhancing the monitoring and prevention of early-onset cancers in these regions is crucial.

Genetic screening has become an indispensable tool due to its emphasis on the prevention of early-stage cancer. For example, current research indicated that breast and ovarian cancer were associated with variants in the *BRCA1* and *BRCA2* genes.^47^ Besides, approximately 10% of CRC cases have been found to be associated with pathogenic variants according to research studies.^20^ Research studies have revealed that these pathogenic variants were detected in 15%–33% of individuals who were diagnosed with CRC before the age of 50, regardless of their family history of the disease.^20^ More importantly, next-generation sequencing has led to improvements in the accessibility and affordability of genetic testing for cancer susceptibility genes. Therefore, genetic screening is expected to have a significant impact on the identification and anticipation of early-onset cancers in the near future.

However, the study still has several limitations due to GBD 2019’s intrinsic drawbacks. First, the accuracy of GBD data was compromised by the quality of cancer registry data in different countries. Thus, the under-reporting and under-diagnosis in undeveloped countries may result in underestimation of the incidences and deaths of early-onset cancer. Second, the increasing trend of early-onset cancer burden is still unclear, which may be related to early screening intervention and early-life exposures. Third, the estimation of risk factor exposure was conducted on data with sparse investigation time nodes and different sources, which may affect influence accuracy and introduce potential measurement bias. Fourth, it is inevitable that implementing a dichotomy at 50 years of age has drawbacks because pathological, molecular and biological characteristics are unlikely to change considerably at that age.

## Conclusions

Our study showed that the global morbidity of early-onset cancer increased from 1990 to 2019, while mortality and DALYs slightly decreased. The rate of incidence, mortality and DALY varied widely across regions, countries and cancer types. The highest-burden regions and cancer types were high-middle and middle SDI regions and early-onset breast cancer, TBL cancer, CRC and stomach cancer, respectively. Dietary risk factors, alcohol use and tobacco consumption were the main risk factors for top early-onset cancers in 2019. Additionally, it is necessary to conduct prospective life-course cohort studies to explore the aetiologies of early-onset cancers. Encouraging a healthy lifestyle, including a healthy diet, the restriction of tobacco and alcohol consumption and appropriate outdoor activity, could reduce the burden of early-onset cancer. It is worth exploring whether early screening and prevention programmes for early-onset cancer should be expanded to include individuals aged 40–44 and 45–49, but further systematic studies and randomised trials are necessary to make a definitive determination.

## Data Availability

Data are available in a public, open access repository. To access the citations for the data utilized in this study, please visit the data input sources tool on the Global Health Data Exchange website (http://ghdx.healthdata.org/gbd-2019/data-input-sources). Complete files containing all GBD 2019 estimates can be obtained from the Global Health Data Exchange website (http://ghdx.healthdata.org/gbd-2019) or downloaded using the Global Health Data Exchange results tool (http://healthdata.org/gbd-results-tool). Reference: GBD 2019 Cancer Risk Factors Collaborators. The global burden of cancer attributable to risk factors, 2010-19: a systematic analysis for the Global Burden of Disease Study 2019. Lancet. 2022;400(10352):563-591. doi:10.1016/S0140-6736(22)01438-6.
