# Supplementary material for: Global trends in incidence, death, burden and risk factors of early-onset cancer from 1990 to 2019
Source: BMJ Oncol. 2023 Sep 5;2(1):e000049. doi: 10.1136/bmjonc-2023-000049 (PMC11235000; doi:10.1136/bmjonc-2023-000049)

Supplementary Methods

Data source, definition of early-onset cancer and risk factors

We extracted metadata on the burden of 29 early-onset cancers at the global, regional and national levels from the GBD 2019 database, which is accessible via the GBD citation tool online (http://ghdx.healthdata.org/gbd-2019) [1]. Twenty-nine early-onset cancers were ascertained from administrative data according to International Classification of Disease version 9 (ICD-9) and 10 (ICD-10) codes, the ICD codes of 29 cancers are shown in Supplementary Table S1. Early-onset cancer was defined as cancer cases diagnosed from 14 to 49 years [2]. In total, 204 countries were divided into five-level regions based on the socio-demographic index (SDI), the SDI comprehensively represents development status in different regions [3]. It was calculated based on lag distributed income per capita, mean years of schooling for individuals 15 years and older, and total fertility rate for individuals younger than 25 years, ranging 0 to 1 [4]. The human development index (HDI) data were obtained at the national level from the World Bank. The GBD database in 2019 estimated the population (including a comprehensive update on fertility and migration), mortality and life expectancy for a total of 990 locations, in 204 countries and territories based on a total of 1250 censuses and 747 location-years of population registry data. In addition, the Bayesian population model was used to reconcile censuses and registry data [1]. We considered morbidity and mortality, defined as the number of incident cases and deaths per 100k individuals. The burden of early-onset

cancer expressed by DALYs was retrieved to represent healthy years lost annually per 100k individuals) [5]. The DALY equation is as follows: $DALYs = years\ lived\ with\ disability\ (YLDs) + years\ of\ life\ lost\ (YLLs)$. YLDs was estimated by diagnosis or treatment, remission, metastatic disseminate, and terminal phase modules, and each module prevalence multiplied by a disability weight was to calculate YLDs. YLLs was calculated by multiplying the number of deaths in the specific age with the corresponding standard life expectancy.

GBD 2019 examined three groups risk factors (environmental and occupational, behavioural, and metabolic), including 23 cancer types and 34 risk factors, with risk factors identified with the World Cancer Research Fund (WCRF) criteria [6]. Behavioral (including dietary risk factors) and metabolic risk factors were examined for their attributable risk to the burden of early-onset cancer in this study. Definitions of these risk factors and method for calculating the proportions of their attribution are described and reported in the previous study [7]. Briefly, to evaluate the burden associated with each risk factor, the GBD 2019 adopted the basic framework developed for comparative risk assessment, including six main steps for each risk–outcome pair [7,8]. First, risk factors with persuasive or likely evidence of a causal connection were identified using the World Cancer Research Fund criteria. For GBD 2019, systematic reviews were revised to ensure that risk factors were appropriately included [7]. Second, existing systematic reviews were updated and meta-analyses of relative risks were performed using GBD's meta-regression-Bayesian, regularised, trimmed

tool in order to estimate relative risks for each risk–outcome pair as a function of exposure. Third, risk factor exposure levels and distributions were modeled with Bayesian meta-regression modelling (DisMod-MR 2.1) or spatiotemporal Gaussian process regression for every age, sex, location, and year combination using data from published studies. Fourth, the theoretical minimum risk exposure level of risk factor was confirmed. Fifth, the population attributable fraction of corresponding risk factor was calculated across age, sex, location, and year, based on the risk function, exposure level and the theoretical minimum risk exposure level. Sixth, mediation was considered for some risk factor to estimate the population attributable fraction [7].

Statistical analysis

The incidence rate, death rate and estimated annual percentage change (EAPC) were used to quantify the epidemic trends of 29 early-onset cancers. EAPC was applied to depict the trends of morbidity and mortality, which has been wildly used in public health studies [9,10]. A regression line was fitted to the natural logarithm of the rates, and the EAPC and its 95% confidence intervals (CIs) were calculated by the linear regression model. The formulas were as follows:

$$y = \alpha + \beta x + \varepsilon$$

$$EAPC = 100 \times (\exp(\beta) - 1)$$

Thereinto, y = ln (rate) and x = calendar year. Meanwhile, we conducted the age-standardization to balance multiple population with different age structures

or for the same population over time to facilitate the comparison between different regions and nations. The age-standardized rate (ASR)/100k population, including age-standardized incidence rate (ASIR) and age-standardized death rate (ASDR), were estimated with the following formula:

$$ASR = \frac{\sum_{i=1}^{A} a_i W_i}{\sum_{i=1}^{A} W_i} \times 100k$$

For above formula, α_i meant the age-specific rate in the ith age group, w meant the number of population (or the weight) in the corresponding i^{th} age group from among the selected reference standard population (global populations from 1990 to 2019 used in this study from GBD) (Table S2), and A meant the number of age groups. The age groups for age-standardized included 15-19 years, 20-24 years, 25-29 years, 30-34 years, 35-39 years, 40-44 years and 45-49 years. We explored the associations between EAPCs and HDI in 2019 using Spearman correlation analysis. Additionally, the Bayesian age-period-cohort (BAPC) model integrating nested Laplace approximations was used to project the morbidity and mortality of the disease burden attributable to early-onset cancer from 2020 to 2030 [11], and the formula was as follow:

$$R_{\mathrm{ijk}} = \mu + \alpha_i\, Age + \beta_j\, Period + \gamma_k$$

Among the parameters of the formula, μ as a constant, R_{ijk} represents the incidence or mortality rate in the ith age group, jth time period, and kth birth cohort. α_i, β_j, and γ_k are the effects of age, time period, and birth cohort. In the BAPC model, the prior probability distribution of time period and birth cohort

effects were the prior information, and the effects of age, period, and cohort through a random walk of different orders were estimated [11]. To avoid over dispersion, an independent random effect, $z_{ij} \sim (0, k_z^{-1})$, was added into the model:

$$R_{\mathrm{ijk}} = \mu + \alpha_i \, Age + \beta_{j+t} \, Period + \gamma_{k+t} \, Cohort + z_{ij+t}$$

Data analysis and graphics were conducted using R 4.2.1 (Lucent Technologies, Jasmine Mountain, USA). *P*-value < 0.05 was considered to be statistically significant.

Reference

1. Global burden of 369 diseases and injuries in 204 countries and territories, 1990-2019: a systematic analysis for the Global Burden of Disease Study 2019. *Lancet* 2020; **396**(10258): 1204-22.
2. Gu WJ, Pei JP, Lyu J, et al. The Burden of Early-Onset Colorectal Cancer and Its Risk Factors from 1990 to 2019: A Systematic Analysis for the Global Burden of Disease Study 2019. *Cancers (Basel)* 2022; **14**(14).
3. Global, regional, and national incidence, prevalence, and years lived with disability for 354 diseases and injuries for 195 countries and territories, 1990-2017: a systematic analysis for the Global Burden of Disease Study 2017. *Lancet* 2018; **392**(10159): 1789-858.
4. Beck S, Wojdyla D, Say L, et al. The worldwide incidence of preterm birth: a systematic review of maternal mortality and morbidity. *Bull World Health Organ* 2010; **88**(1): 31-8.
5. Murray CJ, Lopez AD. Evidence-based health policy--lessons from the Global Burden of Disease Study. *Science* 1996; **274**(5288): 740-3.
6. World Cancer Research Fund AIfC, Research. Food, nutrition, and physical activity, and the prevention
of cancer: a global perspective. *Washington, DC: American Institute for Cancer Research* 2007.
7. Global burden of 87 risk factors in 204 countries and territories, 1990-2019: a systematic analysis for the Global Burden of Disease Study 2019. *Lancet* 2020; **396**(10258): 1223-49.
8. The global burden of cancer attributable to risk factors, 2010-19: a systematic analysis for the Global Burden of Disease Study 2019. *Lancet* 2022; **400**(10352): 563-91.
9. Ou ZJ, Yu DF, Liang YH, et al. Trends in burden of multidrug-resistant tuberculosis in countries, regions, and worldwide from 1990 to 2017: results from the Global Burden of Disease study. *Infect Dis Poverty* 2021; **10**(1): 24.

10. Hankey BF, Ries LA, Kosary CL, et al. Partitioning linear trends in age-adjusted rates. *Cancer Causes Control* 2000; **11**(1): 31-5.
11. Riebler A, Held L. Projecting the future burden of cancer: Bayesian age-period-cohort analysis with integrated nested Laplace approximations. *Biom J* 2017; **59**(3): 531-49.

Table S1. The International Classification of Disease version 9 and 10 codes of 29 early-onset cancers

Cause	ICD10	ICD10 Used in Hospital/Claims Analyses	ICD9	ICD9 Used in Hospital/Claims Analyses
Bladder cancer	C67-C67.9, Z12.6-Z12.79, Z80.52, Z85.51		188-188.9, V10.51, V16.52, V76.3	
Brain and central nervous system cancer	C70-C70.1, C70.9-C72.9, Z85.841-Z85.848, Z86.011		191-191.9	
Breast cancer	C50-C50.629, C50.8-C50.929, Z12.3-Z12.39, Z80.3, Z85.3, Z86.000		174-175.9, V10.3, V16.3	
Cervical cancer	C53-C53.9, Z12.4, Z85.41		180-180.9, V10.41, V72.32	
Colon and rectum cancer	C18-C19.0, C20, C21-C21.8, Z12.1-Z12.13, Z85.03-Z85.048, Z86.010		153-154.9, 209.1-209.17, V10.05-V10.06, V76.41, V76.5-V76.52	
Esophageal cancer	C15-C15.9, Z85.01		150-150.9	
Gallbladder and biliary tract cancer	C23, C24-C24.9		156-156.9	
Hodgkin lymphoma	C81-C81.49, C81.7-C81.79, C81.9-C81.99, Z85.71-Z85.72		201-201.98, V10.72	
Kidney cancer	C64-C64.2, C64.9-C65.9, Z80.51, Z85.52-Z85.54		189-189.1, 189.5-189.6, 209.24	
Larynx cancer	C32-C32.9, Z85.21		161-161.9, V10.21	
Leukemia	C91-C93.7, C93.9-C95.2, C95.7-C95.92, Z80.6, Z85.6		204-208.92, V10.59-V10.69, V16.6	
Lip and oral cavity cancer	C00-C07, C08-C08.9, Z85.81-Z85.810		140-145.9, V76.42	
Liver cancer	C22-C22.4, C22.7-C22.9, Z85.05		155-155.9, V10.07	
Malignant skin melanoma	C43-C43.9, Z85.82-Z85.828		172-172.9	

Mesothelioma	C45-C45.2, C45.7, C45.9			
Multiple myeloma	C88-C90.32		203-203.9	
Nasopharynx cancer	C11-C11.9		147-147.9	
Non-Hodgkin lymphoma	C82-C85.29, C85.7-C86.6, C96-C96.9		200-200.9, 202-202.98	
Non-melanoma skin cancer	C44.01-C44.99	C44.01-C44.92	173-173.99	173.01-173.92
Other malignant neoplasms				
Other pharynx cancer	C09-C10.9, C12-C13.9		146-146.9, 148-148.9	
Ovarian cancer	C56-C56.2, C56.9, Z80.41, Z85.43		183-183.0, 183.8-183.9, V10.43, V16.41	
Pancreatic cancer	C25-C25.9, Z85.07		157-157.9	
Prostate cancer	C61-C61.9, Z12.5, Z80.42, Z85.46		185-185.9, V10.46, V16.42, V76.44	
Stomach cancer	C16-C16.9, Z12.0, Z85.02-Z85.028		151-151.9, 209.23, V10.04	
Testicular cancer	C62-C62.92, Z80.43, Z85.47-Z85.48		186-186.9, V10.47-V10.48, V16.43	
Thyroid cancer	C73, Z85.850		193-193.9	
Tracheal, bronchus, and lung cancer	C33, C34-C34.92, Z12.2, Z80.1-Z80.2, Z85.1-Z85.20		162-162.9, 209.21, V10.1-V10.20, V16.1-V16.2, V16.4-V16.40	
Uterine cancer	C54-C54.3, C54.8-C54.9, Z85.42, Z86.001		182-182.9	

Table S2. The global populations from 1990 to 2019 from Global Burden of Disease database.

Age group	Year	Population (Number)		
		value	95% UI upper	95% UI lower
15 to 19	1990	519603412.1	530680498.1	508300320.3
20 to 24	1990	492675757.3	503896044.6	481582093.4
25 to 29	1990	442843296.3	452298683.7	433270420.5
30 to 34	1990	385628936.3	393643207.2	377634667.2
35 to 39	1990	352742337.2	360428575.9	345093616.7
40 to 44	1990	286291792.8	292117447.4	280385026.7
45 to 49	1990	232409454.8	237083522.7	227751425.8
total	1990	2712194987	2770147980	2654017571
15 to 19	1991	518431189.8	529625669	506685286
20 to 24	1991	497721431.6	509107338.8	485392189.6
25 to 29	1991	455832152.7	466284103.7	444935393.9
30 to 34	1991	391230084.1	399372571.7	382820363.8
35 to 39	1991	361169652.2	369328193	352730424
40 to 44	1991	300417868	306725084.9	293732738.6
45 to 49	1991	235818955.1	240649046.4	230624980.1
total	1991	2760621334	2821092008	2696921376
15 to 19	1992	517752112.3	529645040.2	505664457.5
20 to 24	1992	501355296.6	513963392.1	488822164.2
25 to 29	1992	467066293.3	478885507.3	455232836.3
30 to 34	1992	399751499.4	408609391.4	390772117.8
35 to 39	1992	368327533.8	377433002.9	359401450.8
40 to 44	1992	310706133.5	317936104	303474218.7
45 to 49	1992	244256904	249728441	238590364.2
total	1992	2809215773	2876200879	2741957610
15 to 19	1993	518331320.9	530589982.8	505383791.4
20 to 24	1993	503737367.1	516634043	490381654.2
25 to 29	1993	475498861.8	488308914.1	462208164.3
30 to 34	1993	412123993.8	422123959.6	401912314.7
35 to 39	1993	373316483.4	382820829.4	363520327.1
40 to 44	1993	321665708.5	329832878.7	313297659.4
45 to 49	1993	253832429.9	259866960.1	247418735.3
total	1993	2858506166	2930177568	2784122646
15 to 19	1994	520867528.1	534036366.2	507564349.3
20 to 24	1994	505044168.7	519205490	490995146.2
25 to 29	1994	481852637	496002910.9	467594339.3
30 to 34	1994	426649539.2	438369965	415004429.2
35 to 39	1994	376959573.7	387075630.1	366693145.6
40 to 44	1994	331572772.1	340918924.3	322309246.9
45 to 49	1994	266054126.5	273106002.2	258906637.1
total	1994	2909000345	2988715289	2829067294

15 to 19	1995	525627131.5	539810121.8	511786579.1
20 to 24	1995	505533844.6	520435296.8	490792209.9
25 to 29	1995	487044565.9	502559413.8	471506826
30 to 34	1995	441148313.6	454486155.5	427714659.1
35 to 39	1995	380779750.6	391656066.8	370221444.8
40 to 44	1995	344801865.7	355292420.6	334523992.1
45 to 49	1995	276148943.3	283980606.7	268285253.1
total	1995	2961084415	3048220082	2874830964
15 to 19	1996	532746680	546959076.7	518596772.5
20 to 24	1996	505332329.5	519527208.4	491141002.5
25 to 29	1996	492230166	507261276.7	477324491.6
30 to 34	1996	453915291.6	467610171.6	440183343.3
35 to 39	1996	386716603.9	397485130.6	376108907.7
40 to 44	1996	352517513.3	362937302.1	342178577.8
45 to 49	1996	289621233.1	297893216.6	281474561.1
total	1996	3013079817	3099673383	2927007656
15 to 19	1997	541569409.9	555217526.3	527501869.3
20 to 24	1997	505471466.9	518641456.4	491842204.4
25 to 29	1997	496413797.2	510280881	481685530.6
30 to 34	1997	464801665.8	478011948.4	450670088.8
35 to 39	1997	395709386.2	405717465.2	385116111.9
40 to 44	1997	359075632.2	368936016.8	348777035.7
45 to 49	1997	299324118	307157744	290953335.5
total	1997	3062365476	3143963038	2976546176
15 to 19	1998	551433118.6	564737008	538525999.1
20 to 24	1998	506664550.5	519038997.2	494424129.5
25 to 29	1998	499486643.4	512639674.3	486328469.7
30 to 34	1998	472793184.4	485686479.5	459824502.3
35 to 39	1998	408547814.3	418487971.6	398427069.2
40 to 44	1998	363613013.4	372858162.5	354162295.9
45 to 49	1998	309743148.6	317472961.4	301744625.7
total	1998	3112281473	3190921254	3033437091
15 to 19	1999	561765793.9	573840530.8	549514415.4
20 to 24	1999	509518057.1	520480382.6	498385100.7
25 to 29	1999	501514045.8	513289855	489588813.4
30 to 34	1999	478748391	490504854.8	466591931.9
35 to 39	1999	423428022.5	433120590.9	413526445.3
40 to 44	1999	366896556.8	375162052.1	358369381.9
45 to 49	1999	319090325.7	326456829.8	311442125.9
total	1999	3160961193	3232855096	3087418214
15 to 19	2000	572711000.5	584570505.3	560653361.1
20 to 24	2000	514381490.6	524841210.7	503645582
25 to 29	2000	502647111.1	513618289.9	491343042.8
30 to 34	2000	483765873.5	494906930.3	472250617

35 to 39	2000	438184947.2	447954574.6	427994194.1
40 to 44	2000	370461890.2	378141421.5	362426860.2
45 to 49	2000	331717054.9	338950651.7	324152892.5
total	2000	3213869368	3282983584	3142466550
15 to 19	2001	584931684.8	597054315.5	572619149.4
20 to 24	2001	521426557	531907452.8	510553254.1
25 to 29	2001	502778255.8	513825110.4	491695869.7
30 to 34	2001	489107892	500472869.5	477631548.2
35 to 39	2001	450910563.8	461444044.9	440234900.5
40 to 44	2001	376209318	384124036.5	368094930.6
45 to 49	2001	339226508.3	347002229.8	331428893
total	2001	3264590780	3335830059	3192258546
15 to 19	2002	597719508.8	611699883	584089197.3
20 to 24	2002	530224078.1	542057890.2	518438411.4
25 to 29	2002	503038577.1	514865407.1	491191485.6
30 to 34	2002	493665792.2	506053212.6	480975927.6
35 to 39	2002	461626087.8	473611387.3	449411890.2
40 to 44	2002	385026319.7	393866389.2	376014046
45 to 49	2002	345682300.8	354306020.6	337032389.8
total	2002	3316982664	3396460190	3237153348
15 to 19	2003	609967034.5	625187073.2	594795523.5
20 to 24	2003	540129344.8	552899935.6	527393988.4
25 to 29	2003	504273518.2	516656822.7	491991160.6
30 to 34	2003	497207785.2	510054168.9	483941892.8
35 to 39	2003	469474307.6	482259520.7	456384977.4
40 to 44	2003	397639384.8	407361471	387558433.6
45 to 49	2003	350246132.1	359252998.1	341021192.2
total	2003	3368937507	3453671990	3283087168
15 to 19	2004	620429802	637255911.9	603658073.6
20 to 24	2004	550457476.5	564531386.4	536483664.2
25 to 29	2004	507068043.7	520080083.6	493802524.9
30 to 34	2004	499698985.9	513656569.5	485432029.9
35 to 39	2004	475388405.9	489074818.6	461195035.5
40 to 44	2004	412265186.6	423386424.4	400795939.6
45 to 49	2004	353640784.9	362892312.4	343999007.9
total	2004	3418948685	3510877507	3325366276
15 to 19	2005	627746727.5	645910118.8	610174954.4
20 to 24	2005	561336218.8	576549848.6	546265825.3
25 to 29	2005	511675130.1	525377586.9	497735936.5
30 to 34	2005	501153714.4	515877113.7	486530733.2
35 to 39	2005	480401874.4	495932256.1	465451537.8
40 to 44	2005	426776464.4	439847183.5	414157740.4
45 to 49	2005	357325169.5	367414504.9	347377902.8
total	2005	3466415299	3566908613	3367694630

15 to 19	2006	630491830.2	649229178.7	612304102.7
20 to 24	2006	573821690	590337130.4	557843963.4
25 to 29	2006	518507526.2	533235102.8	504281929.5
30 to 34	2006	501610073	516516227.9	486810121.4
35 to 39	2006	485831409.2	500998980.5	470589625.1
40 to 44	2006	439314572.4	452820313.7	425661215.2
45 to 49	2006	363172616.8	372941316.5	352857966.5
total	2006	3512749718	3616078251	3410348924
15 to 19	2007	629442031.1	646295218.8	612499765.7
20 to 24	2007	587350039.5	602610301.3	571694341
25 to 29	2007	527232718.6	540698208.5	513836038.1
30 to 34	2007	502257145.1	515603020.5	488928549.6
35 to 39	2007	490575061.2	504677852.9	476275453.3
40 to 44	2007	449989584.1	462861877.1	436648140.7
45 to 49	2007	372071189.2	381575480.7	362348132.8
total	2007	3558917769	3654321960	3462230421
15 to 19	2008	625501004.8	640328892.5	610271163.4
20 to 24	2008	600238491.1	614894853	585371297
25 to 29	2008	536869167.1	549331310.9	524396861.2
30 to 34	2008	503719503.2	515663033.6	491411781.7
35 to 39	2008	494258405.8	507040715.4	481382118.5
40 to 44	2008	457844356.2	469781842.6	445508393.3
45 to 49	2008	384677767.3	393677275.7	375189293.5
total	2008	3603108696	3690717924	3513530909
15 to 19	2009	620182568.5	634270507.2	606911715
20 to 24	2009	610877828	625020486.3	597067578.1
25 to 29	2009	546596215.5	558505271.1	534922972.6
30 to 34	2009	506437833.9	517785005.9	495277724.2
35 to 39	2009	496779149.6	508371538.2	485216706.2
40 to 44	2009	463763007.9	475093496.9	452442257.9
45 to 49	2009	399220279.3	408242252.7	390258823
total	2009	3643856883	3727288558	3562097777
15 to 19	2010	614905315.7	627682461.7	602455449.2
20 to 24	2010	618197109.4	631506402.2	605083502
25 to 29	2010	556937766.6	568257932.8	545572491.3
30 to 34	2010	510863945.2	521349257.2	500365347.5
35 to 39	2010	498339906.9	509133871.4	487396308.4
40 to 44	2010	468870865.8	479379365.3	458374062.7
45 to 49	2010	413683324.9	422481082.2	404810524.7
total	2010	3681798235	3759790373	3604057686
15 to 19	2011	611004326.6	623438739	598700918.6
20 to 24	2011	621004896	634923308	607743528.1
25 to 29	2011	568854206.8	580965354.5	557091388.1
30 to 34	2011	517307082.1	528239970.6	506892697.2

35 to 39	2011	498831665.9	510054449.4	487894843.3
40 to 44	2011	474428276.4	486099984.6	463196920.3
45 to 49	2011	426164043	436631599.7	416101956.1
total	2011	3717594497	3800353406	3637622252
15 to 19	2012	608599249.6	621623994.2	595771283.9
20 to 24	2012	619902721.6	634185027	605447816.4
25 to 29	2012	581549897.1	594815688.9	568439583.8
30 to 34	2012	525336993.4	536676719.7	513823571.9
35 to 39	2012	499285168.6	510948734.5	487300751
40 to 44	2012	479203061.1	491142019.8	466882896.6
45 to 49	2012	436828062.3	448305890.5	425072911.3
total	2012	3750705154	3837698075	3662738815
15 to 19	2013	607448157.1	621183643.3	593654916.9
20 to 24	2013	616102175.2	631308923.7	601259565.3
25 to 29	2013	593849144.7	608648508.2	579034752.6
30 to 34	2013	534426345	546818918.7	521774076.2
35 to 39	2013	500574699.6	512891450.5	488059434.1
40 to 44	2013	482984401.2	495903478.9	469744193
45 to 49	2013	444807473.5	457482128.2	432033962.4
total	2013	3780192396	3874237052	3685560901
15 to 19	2014	607279470.4	621922154.5	592278065.3
20 to 24	2014	611104444.2	626705120	595244495.2
25 to 29	2014	604446564.9	620120233.1	587965973.5
30 to 34	2014	544030593.2	557351969.7	530070906.2
35 to 39	2014	503338810.4	515992995.6	490067180
40 to 44	2014	485771436.6	499226313.9	471956896.2
45 to 49	2014	450968489.8	464249991.4	437468017
total	2014	3806939810	3905568778	3705051533
15 to 19	2015	608218087.6	623622681.7	592376348.4
20 to 24	2015	606112142.2	622744675.2	589837526.6
25 to 29	2015	612012137	628815900.7	594577158.7
30 to 34	2015	554407501.9	569269450.1	539122605.6
35 to 39	2015	507892129.9	521330451.5	493847217.8
40 to 44	2015	487612069.2	501695247.2	473185523.3
45 to 49	2015	456297755.8	470321181.9	441780065.7
total	2015	3832551824	3937799588	3724726446
15 to 19	2016	610093992.1	626604794	592844022.2
20 to 24	2016	602557005.1	620090585.6	585352846
25 to 29	2016	614984761.6	633145816.8	596184994.3
30 to 34	2016	566303551.5	582918522.1	549474113.7
35 to 39	2016	514363495.7	529328722.3	499336052.5
40 to 44	2016	488320878	503226274.7	473000968.3
45 to 49	2016	462025193.2	477592660.6	446919426.9
total	2016	3858648877	3972907376	3743112424

15 to 19	2017	612577366.2	630504939	594845461.4
20 to 24	2017	600599320.1	619121599.8	582944860.2
25 to 29	2017	614004651	634481806.8	594512501.1
30 to 34	2017	578913277.4	598037280.6	560584333.7
35 to 39	2017	522346015.5	538808826.4	506174465.6
40 to 44	2017	488978593.4	505035158.4	472768963.1
45 to 49	2017	466917924.3	483082675.1	449912324.5
total	2017	3884337148	4009072286	3761742910
15 to 19	2018	615767495.4	633590404.6	596886100.5
20 to 24	2018	599885085.9	618055364.1	581519599.2
25 to 29	2018	610283133.9	630271788.3	590563763.3
30 to 34	2018	591133850.7	610761189.7	571645816.6
35 to 39	2018	531387014.5	548149258.5	514538370.3
40 to 44	2018	490465267.1	506372984.1	474366524.5
45 to 49	2018	470841955.4	487805016.7	454027560.5
total	2018	3909763803	4035006006	3783547735
15 to 19	2019	619540978.1	639410643.1	600449626
20 to 24	2019	600144464.9	619566711.2	581021870.5
25 to 29	2019	605470102.4	626202183.3	584989358.8
30 to 34	2019	601732453.7	623410373.9	580098350.7
35 to 39	2019	540976121.5	559170147.5	522534368.2
40 to 44	2019	493443132.4	510303392	476222491.8
45 to 49	2019	473801065.3	491190698.2	455039768.8
total	2019	3935108318	4069254149	3800355835

Table S3. Incidence and corresponding EAPC of 29 early-onset cancer types in 1990 and 2019.

Early-onset cancer types	1990 Incidence		2019 Incidence		1990-2019 Incidence
	Cases No. (95% UI)	Rate per 100k No. (95% UI)	Cases No. (95% UI)	Rate per 100k No. (95% UI)	EAPC No. (95% CI)
Bladder cancer	18851.7 (17877.6-19937)	0.7 (0.7-0.7)	38922.2 (34991.5-43223.9)	1 (0.9-1.1)	1 (0.92, 1.08)
Brain and central nervous system cancer	54922.8 (47514.5-66711.4)	2 (1.8-2.5)	102687.2 (80889.9-115093.7)	2.6 (2.1-2.9)	0.8 (0.74, 0.86)
Breast cancer	260836.3 (250473.6-271710.5)	9.6 (9.2-10)	537979.6 (490640.8-588711.2)	13.7 (12.5-15)	1.01 (0.9, 1.12)
Cervical cancer	160770.2 (141427.6-186580.3)	5.9 (5.2-6.9)	256902.2 (215920.8-289544.9)	6.5 (5.5-7.4)	0.28 (0.22, 0.34)
Colorectal cancer	94707.1 (90420.5-99416.4)	3.5 (3.3-3.7)	225736 (207658-246755.7)	5.7 (5.3-6.3)	1.73 (1.65, 1.8)
Esophageal cancer	32454.9 (25094.2-36124.1)	1.2 (0.9-1.3)	40818.9 (36352.6-46025.9)	1 (0.9-1.2)	-0.86 (-1.25, -0.48)
Gallbladder and biliary tract cancer	9006.6 (8136.5-10218.2)	0.3 (0.3-0.4)	13722.5 (11736.3-15194.5)	0.3 (0.3-0.4)	0.18 (0.1, 0.26)
Hodgkin lymphoma	33409.6 (26787.6-36176.5)	1.2 (1-1.3)	43574.3 (39046.3-52320.7)	1.1 (1-1.3)	-0.47 (-0.54, -0.41)
Kidney cancer	23875.5 (22848.6-24856.1)	0.9 (0.8-0.9)	57999.5 (52884.9-63376.5)	1.5 (1.3-1.6)	1.75 (1.62, 1.87)
Larynx cancer	16296.6 (15544.1-17094.1)	0.6 (0.6-0.6)	21549.1 (19780.8-23591)	0.5 (0.5-0.6)	-0.71 (-0.84, -0.58)
Leukemia	109042 (96043.9-120420.4)	4 (3.5-4.4)	155420.8 (139380.4-169956.2)	3.9 (3.5-4.3)	-0.25 (-0.33, -0.16)
Lip and oral cavity cancer	39319.9 (37399.9-41418.2)	1.4 (1.4-1.5)	74344.2 (66884.1-81548.6)	1.9 (1.7-2.1)	0.76 (0.69, 0.83)
Liver cancer	88117.2 (76723.3-101492.9)	3.2 (2.8-3.7)	78299.4 (68668-89097.7)	2 (1.7-2.3)	-2.88 (-3.46, -2.3)
Malignant skin melanoma	38988.2 (29130.2-44281.6)	1.4 (1.1-1.6)	72968.4 (59581.3-91447.6)	1.9 (1.5-2.3)	0.68 (0.51, 0.84)
Mesothelioma	2615.4 (1948.1-3556.7)	0.1 (0.1-0.1)	3740.3 (3161.6-4272.1)	0.1 (0.1-0.1)	-0.32 (-0.43, -0.21)
Multiple myeloma	5277.4 (4704.1-6325)	0.2 (0.2-0.2)	10740.8 (8964.2-12044.2)	0.3 (0.2-0.3)	0.94 (0.76, 1.13)
Nasopharynx cancer	25238.2 (22682.8-27620.8)	0.9 (0.8-1)	67915.2 (59442.4-77614.4)	1.7 (1.5-2)	2.28 (2.1, 2.47)
Non-Hodgkin lymphoma	46087.4 (42095.1-51252.4)	1.7 (1.6-1.9)	81586.8 (74019-90533.3)	2.1 (1.9-2.3)	0.51 (0.42, 0.6)
Non-melanoma skin cancer	238054.1 (186397-300376.3)	8.8 (6.9-11.1)	486545 (401341.6-586406.2)	12.4 (10.2-14.9)	1.59 (1.21, 1.98)

Other malignant neoplasms	113396.4 (100921.9-123848)	4.2 (3.7-4.6)	214565.8 (195583-232245.1)	5.5 (5-5.9)	0.89 (0.85, 0.92)
Other pharynx cancers	13257.5 (12364.8-14312.9)	0.5 (0.5-0.5)	26987.7 (24143.1-29674.5)	0.7 (0.6-0.8)	0.9 (0.78, 1.01)
Ovarian cancer	39872 (34417.7-48766.4)	1.5 (1.3-1.8)	79671.9 (68572.8-90845)	2 (1.7-2.3)	0.91 (0.82, 1)
Pancreatic cancer	17479.4 (16587.8-18435.7)	0.6 (0.6-0.7)	36851.2 (33756.6-40329.9)	0.9 (0.9-1)	1.14 (1.04, 1.24)
Prostate cancer	7831.4 (6374.4-9087.1)	0.3 (0.2-0.3)	22581.2 (19175.2-27761)	0.6 (0.5-0.7)	2.23 (1.97, 2.49)
Stomach cancer	125974.3 (118139.9-133955.5)	4.6 (4.4-4.9)	144321.2 (130472.9-158788.9)	3.7 (3.3-4)	-0.84 (-0.99, -0.7)
Testicular cancer	34332.8 (27953.8-37230.1)	1.3 (1-1.4)	72597.2 (64977.7-81950)	1.8 (1.7-2.1)	1.23 (1.18, 1.27)
Thyroid cancer	35055.2 (31179.3-37947)	1.3 (1.1-1.4)	91032.5 (80018.5-99250.1)	2.3 (2-2.5)	1.96 (1.76, 2.15)
Tracheal, bronchus, and lung cancer	106306.3 (100111.4-113467.8)	3.9 (3.7-4.2)	136946.2 (123422.3-150290.4)	3.5 (3.1-3.8)	-0.7 (-0.9, -0.5)
Uterine cancer	30231.6 (25409.9-32988)	1.1 (0.9-1.2)	63585.2 (54747.7-71083)	1.6 (1.4-1.8)	1.32 (1.03, 1.62)

Abbreviations: EAPC, estimated annual percentage change; UI, uncertainty interval; CI, confidence interval.

Table S4. Death and corresponding EAPC of 29 early-onset cancer types in 1990 and 2019.

Early-onset cancer types	1990 Death		2019 Death		1990-2019 Death
	**Cases No. (95% UI)**	**Rate per 100k No. (95% UI)**	**Cases No. (95% UI)**	**Rate per 100k No. (95% UI)**	**EAPC No. (95% CI)**
Bladder cancer	5598.6 (5227.7-6015.6)	0.2 (0.2-0.2)	7784 (7090.9-8593.1)	0.2 (0.2-0.2)	-0.42 (-0.53, -0.32)
Brain and central nervous system cancer	37501 (31997.3-45876.4)	1.4 (1.2-1.7)	55346.1 (43753.7-61463.2)	1.4 (1.1-1.6)	-0.09 (-0.19, 0.01)
Breast cancer	86957.8 (82562.6-92106.1)	3.2 (3-3.4)	136448.7 (124894.9-149068.5)	3.5 (3.2-3.8)	0 (-0.12, 0.12)
Cervical cancer	58081.3 (50008.7-68379)	2.1 (1.8-2.5)	76549.5 (64144.3-86516.1)	1.9 (1.6-2.2)	-0.42 (-0.5, -0.34)
Colorectal cancer	50436.5 (47475-53367.8)	1.9 (1.8-2)	86545.6 (80162-93431.1)	2.2 (2-2.4)	0.48 (0.4, 0.55)
Esophageal cancer	28632.6 (22111.7-31956.5)	1.1 (0.8-1.2)	32955.9 (29538-37482.2)	0.8 (0.8-1)	-1.19 (-1.57, -0.8)

Gallbladder and biliary tract cancer	6664.5 (5955.8-7687.5)	0.2 (0.2-0.3)	9761.2 (8321.2-10715)	0.2 (0.2-0.3)	0.05 (-0.03, 0.13)
Hodgkin lymphoma	11982.8 (9385-13204.9)	0.4 (0.3-0.5)	11183.8 (9517.9-13128.8)	0.3 (0.2-0.3)	-1.73 (-1.83, -1.63)
Kidney cancer	6845.8 (6485.6-7172.3)	0.3 (0.2-0.3)	12957.9 (11916.3-14161.8)	0.3 (0.3-0.4)	0.81 (0.7, 0.92)
Larynx cancer	10561.8 (9928.2-11154.6)	0.4 (0.4-0.4)	11834 (10813.4-13097.7)	0.3 (0.3-0.3)	-1.28 (-1.42, -1.15)
Leukemia	68426.4 (59766.5-75746.6)	2.5 (2.2-2.8)	69298.4 (62141-75429.3)	1.8 (1.6-1.9)	-1.44 (-1.54, -1.35)
Lip and oral cavity cancer	17492.9 (16405.1-18751.3)	0.6 (0.6-0.7)	31258.4 (27932.4-34762.2)	0.8 (0.7-0.9)	0.58 (0.5, 0.66)
Liver cancer	78416.1 (67900.6-90532.2)	2.9 (2.5-3.3)	60602.5 (53310-68336.1)	1.5 (1.4-1.7)	-3.39 (-4, -2.77)
Malignant skin melanoma	7892 (6189.7-9560.7)	0.3 (0.2-0.4)	9880.3 (8113-12023.4)	0.3 (0.2-0.3)	-0.78 (-0.89, -0.68)
Mesothelioma	1820.9 (1377-2457.6)	0.1 (0.1-0.1)	2707.8 (2318.1-3058.3)	0.1 (0.1-0.1)	-0.14 (-0.24, -0.03)
Multiple myeloma	3539.4 (3127.3-4353.6)	0.1 (0.1-0.2)	6418.1 (5369.8-7111.9)	0.2 (0.1-0.2)	0.52 (0.36, 0.67)
Nasopharynx cancer	16563.7 (14922.1-18246.7)	0.6 (0.6-0.7)	17237.5 (15774.7-18851.4)	0.4 (0.4-0.5)	-1.44 (-1.57, -1.3)
Non-Hodgkin lymphoma	25306.5 (23603.2-27119)	0.9 (0.9-1)	38451.3 (35770.2-41630.3)	1 (0.9-1.1)	0 (-0.08, 0.08)
Non-melanoma skin cancer	2517.8 (2321.6-2736.7)	0.1 (0.1-0.1)	3748.6 (3450-4020.7)	0.1 (0.1-0.1)	0.07 (-0.03, 0.17)
Other malignant neoplasms	56333.1 (50107.6-60578.5)	2.1 (1.8-2.2)	83689 (75459.7-91529.7)	2.1 (1.9-2.3)	-0.06 (-0.1, -0.01)
Other pharynx cancers	9215.2 (8498.4-10176.1)	0.3 (0.3-0.4)	16843.5 (14816.9-18804.9)	0.4 (0.4-0.5)	0.56 (0.46, 0.66)
Ovarian cancer	15138.9 (12927.3-18976.1)	0.6 (0.5-0.7)	27759.2 (23923-31721.7)	0.7 (0.6-0.8)	0.59 (0.49, 0.69)
Pancreatic cancer	15285.8 (14453.1-16200.3)	0.6 (0.5-0.6)	32004 (29401.6-34968.4)	0.8 (0.7-0.9)	1.11 (1.01, 1.21)
Prostate cancer	2110.1 (1706.7-2332.4)	0.1 (0.1-0.1)	3574.1 (3008.8-4281.9)	0.1 (0.1-0.1)	0.33 (0.23, 0.44)
Stomach cancer	97382.7 (90808.2-104061.7)	3.6 (3.3-3.8)	87333.5 (79904.3-95000.1)	2.2 (2-2.4)	-1.78 (-1.99, -1.58)
Testicular cancer	4917.3 (4639.3-5178.3)	0.2 (0.2-0.2)	6867.7 (6306.5-7457.3)	0.2 (0.2-0.2)	-0.19 (-0.28, -0.1)
Thyroid cancer	3795.8 (3246-4304)	0.1 (0.1-0.2)	5925.5 (5262.9-6473.7)	0.2 (0.1-0.2)	0.11 (0, 0.22)
Tracheal, bronchus, and lung cancer	91573.6 (85931-98019.2)	3.4 (3.2-3.6)	110729.4 (101076.6-120624.3)	2.8 (2.6-3.1)	-0.94 (-1.14, -0.74)
Uterine cancer	5878.3 (4443.6-6749.3)	0.2 (0.2-0.2)	7120.6 (5825.5-7914.1)	0.2 (0.1-0.2)	-0.83 (-1.13, -0.52)

Abbreviations: EAPC, estimated annual percentage change; UI, uncertainty interval; CI, confidence interval.

Table S5. Disability-adjusted life years (DALYs) and corresponding EAPC of 29 early-onset cancers in 1990 and 2019.

Early-onset cancer types	1990 DALYs		2019 DALYs		1990-2019 DALYs
	Cases No. (95% UI)	Rate per 100k No. (95% UI)	Cases No. (95% UI)	Rate per 100k No. (95% UI)	EAPC No. (95% CI)
Bladder cancer	279920.4 (260428.6-301617.8)	10.3 (9.6-11.1)	390740.7 (357671.3-429267.4)	9.9 (9.1-10.9)	-0.41 (-0.51, -0.31)
Brain and central nervous system cancer	2027034.2 (1731600.3-2486867.8)	74.7 (63.8-91.7)	2917783.9 (2305065.6-3247570.8)	74.1 (58.6-82.5)	-0.17 (-0.26, -0.08)
Breast cancer	4327460.9 (4102075.4-4590967.7)	159.6 (151.2-169.3)	6832262.2 (6219746.4-7431400.4)	173.6 (158.1-188.8)	0.02 (-0.1, 0.14)
Cervical cancer	2913442.6 (2504657.5-3437397.7)	107.4 (92.3-126.7)	3801241.7 (3175065.5-4285528.7)	96.6 (80.7-108.9)	-0.47 (-0.55, -0.38)
Colorectal cancer	2516720.9 (2368905.6-2663625.4)	92.8 (87.3-98.2)	4259922 (3942849.9-4590979.2)	108.3 (100.2-116.7)	0.42 (0.35, 0.49)
Esophageal cancer	1327637 (1026867.4-1476512.8)	49 (37.9-54.4)	1516319 (1361708.2-1720376.2)	38.5 (34.6-43.7)	-1.22 (-1.59, -0.84)
Gallbladder and biliary tract cancer	315792.5 (282111.5-364221.4)	11.6 (10.4-13.4)	456165.2 (388506.3-500980.1)	11.6 (9.9-12.7)	-0.01 (-0.09, 0.07)
Hodgkin lymphoma	696643.2 (547556.6-767547.2)	25.7 (20.2-28.3)	650560.1 (554149-764527.6)	16.5 (14.1-19.4)	-1.71 (-1.81, -1.61)
Kidney cancer	341994.9 (323772.6-359870.6)	12.6 (11.9-13.3)	646339.9 (594759.4-706070)	16.4 (15.1-17.9)	0.82 (0.72, 0.93)
Larynx cancer	496236.2 (467216.1-524227.7)	18.3 (17.2-19.3)	555495.2 (508685.9-611840.7)	14.1 (12.9-15.5)	-1.29 (-1.42, -1.15)
Leukemia	3984968.1 (3463513.2-4416809.9)	146.9 (127.7-162.9)	3913497.7 (3502797.6-4267249.9)	99.5 (89-108.4)	-1.54 (-1.64, -1.45)
Lip and oral cavity cancer	858787.6 (804949.5-919796.6)	31.7 (29.7-33.9)	1527398 (1366591.5-1697046.4)	38.8 (34.7-43.1)	0.55 (0.48, 0.63)
Liver cancer	3826682 (3314993-4420259.5)	141.1 (122.2-163)	2898076.8 (2554655.9-3261451.2)	73.6 (64.9-82.9)	-3.46 (-4.06, -2.86)
Malignant skin melanoma	415705.6 (327380.4-506830.2)	15.3 (12.1-18.7)	524345.1 (427761.5-639655.8)	13.3 (10.9-16.3)	-0.74 (-0.84, -0.65)
Mesothelioma	91409.1 (67711.5-125083.1)	3.4 (2.5-4.6)	132402.6 (112421-150038.2)	3.4 (2.9-3.8)	-0.25 (-0.35, -0.14)
Multiple myeloma	169068.4 (150052.6-209555.5)	6.2 (5.5-7.7)	306838.9 (256087.6-340491.4)	7.8 (6.5-8.7)	0.51 (0.35, 0.67)
Nasopharynx cancer	844065.6 (760336.6-928015.4)	31.1 (28-34.2)	874186.4 (804883.7-952901.8)	22.2 (20.5-24.2)	-1.47 (-1.61, -1.33)
Non-Hodgkin lymphoma	1392131.7 (1294870.8-1494921)	51.3 (47.7-55.1)	2066163.5 (1924546.4-2237846.4)	52.5 (48.9-56.9)	-0.08 (-0.16, -0.01)
Non-melanoma skin cancer	128327.7 (118778.6-139250.1)	4.7 (4.4-5.1)	187302.7 (172963.6-200586.2)	4.8 (4.4-5.1)	-0.01 (-0.1, 0.08)

Other malignant neoplasms	3194375 (2838849.1-3437705)	117.8 (104.7-126.7)	4682788.6 (4222113.3-5113396.2)	119 (107.3-129.9)	-0.1 (-0.14, -0.05)
Other pharynx cancers	436024.6 (401114.7-484058.5)	16.1 (14.8-17.8)	795774.7 (700405.9-888018.6)	20.2 (17.8-22.6)	0.55 (0.45, 0.65)
Ovarian cancer	760870.4 (645441.4-960212.7)	28.1 (23.8-35.4)	1379355.3 (1183284.2-1574161.6)	35.1 (30.1-40)	0.55 (0.45, 0.64)
Pancreatic cancer	723474.5 (682646.9-767793.3)	26.7 (25.2-28.3)	1489527.7 (1368667.3-1627198.3)	37.9 (34.8-41.4)	1.04 (0.95, 1.13)
Prostate cancer	105476.6 (84960.1-115605.9)	3.9 (3.1-4.3)	182131.6 (154399-217655.1)	4.6 (3.9-5.5)	0.41 (0.3, 0.51)
Stomach cancer	4743785.3 (4423216.2-5063886)	174.9 (163.1-186.7)	4206556.7 (3853720.9-4566426.9)	106.9 (97.9-116)	-1.84 (-2.04, -1.64)
Testicular cancer	297954.5 (280555.7-314734.3)	11 (10.3-11.6)	424469.8 (388485.7-462990)	10.8 (9.9-11.8)	-0.11 (-0.2, -0.02)
Thyroid cancer	215255.2 (182182.9-244090.3)	7.9 (6.7-9)	347975.8 (306330.9-384509.9)	8.8 (7.8-9.8)	0.23 (0.12, 0.34)
Tracheal, bronchus, and lung cancer	4308319.8 (4042416-4611324.6)	158.8 (149-170)	5147912.6 (4703827.5-5611862.2)	130.8 (119.5-142.6)	-1 (-1.19, -0.8)
Uterine cancer	295925.1 (223759.1-340039.8)	10.9 (8.3-12.5)	365187 (300719.7-406309.7)	9.3 (7.6-10.3)	-0.76 (-1.05, -0.47)

Abbreviations: EAPC, estimated annual percentage change; UI, uncertainty interval; CI, confidence interval.

Table S6. Incidence and death of early-onset breast cancer, TBL cancer, CRC and stomach cancer by region and sociodemographic index level in 1990 and 2019.

Cancers	SDI regions	1990 Incidence		2019 Incidence		1990 Death		2019 Death	
		Cases (95% UI)	ASR per 100k (95% UI)	Cases (95% UI)	ASR per 100k (95% UI)	Cases (95% UI)	ASR per 100k (95% UI)	Cases (95% UI)	ASR per 100k (95% UI)
Breast cancer	Global	260836.3 (250473.6-271710.5)	11.2 (11.2, 11.3)	537979.6 (490640.8-588711.2)	13.2 (13.1, 13.2)	86957.8 (82562.6-92106.1)	3.7 (3.7, 3.8)	136448.7 (124894.9-149068.5)	3.3 (3.3, 3.4)
	Low SDI	9363.7 (8001.9-10797.6)	5.2 (5.1, 5.3)	32559.2 (27874.8-37975.8)	7.6 (7.5, 7.7)	6211.2 (5321.9-7235.5)	3.5 (3.4, 3.6)	17240.6 (14793.9-20021.6)	4.1 (4, 4.1)
	Low-middle SDI	26776.6 (23729.1-30345.5)	6.1 (6, 6.2)	83499.4 (72485-95330.3)	9.6 (9.6, 9.7)	15570.4 (13780.3-17711.6)	3.6 (3.5, 3.6)	35253.3 (30234.7-40788.5)	4.1 (4, 4.1)
	Middle SDI	52827.9 (48220.2-57995.7)	7.3 (7.2, 7.3)	167017.6 (147164.1-187948)	12.3 (12.2, 12.3)	23883.3 (22021.3-26229.8)	3.3 (3.3, 3.4)	44643.9 (39988.6-49854.2)	3.3 (3.2, 3.3)
	High-middle SDI	68200.6 (65207.6-71583)	12.4 (12.4, 12.5)	134512.5 (119061.7-151755.1)	15.4 (15.3, 15.5)	21200.8 (20171.9-22417)	3.9 (3.8, 3.9)	24208 (22152-26662)	2.8 (2.7, 2.8)
	High SDI	103495.7 (100980.7-105995.3)	22.9 (22.8, 23.1)	120000.7 (106168.2-134790.7)	21.1 (21, 21.2)	20026.5 (19713.9-20347.4)	4.4 (4.4, 4.5)	14976.3 (14320.5-15639.6)	2.6 (2.6, 2.7)
	High-income Asia Pacific	13747.9 (12553.4-15103)	13.5 (13.3, 13.7)	20642.6 (17076.9-24966.4)	18.4 (18.2, 18.7)	2497 (2441.6-2555.3)	2.4 (2.4, 2.5)	2237.2 (2117.6-2376.8)	2 (1.9, 2)

	Central Asia	3310.1 (3141.5-3491.5)	13.1 (12.6, 13.6)	6164.1 (5336.9-7078.8)	12.8 (12.5, 13.1)	1265.5 (1213.4-1323.7)	5.1 (4.8, 5.4)	1816.9 (1584.2-2085)	3.8 (3.6, 4)
	East Asia	34620.3 (27994.9-42038.5)	6 (6, 6.1)	112508.6 (86987.1-140589.7)	12.2 (12.1, 12.2)	13253.6 (10745.4-16007.2)	2.3 (2.3, 2.4)	17176.3 (13618-21402)	1.8 (1.8, 1.9)
	South Asia	21947.2 (18713.6-25110.8)	4.9 (4.9, 5)	78582.1 (64308.5-94610.5)	8.7 (8.6, 8.7)	13489.2 (11514.3-15394.2)	3.1 (3, 3.1)	35091.7 (28704.5-42380.8)	3.9 (3.9, 3.9)
	Southeast Asia	21600 (18378.9-25278.5)	11.4 (11.2, 11.5)	59060.6 (49804.5-69732.6)	15.6 (15.5, 15.7)	11368.3 (9772.4-13411.8)	6.1 (5.9, 6.2)	20994.5 (17773.5-24976.1)	5.5 (5.5, 5.6)
	Australasia	2775.8 (2597.1-2951)	25 (24.1, 26)	3720.3 (2886.9-4754.8)	23.4 (22.7, 24.2)	562.2 (540.5-583.5)	5.1 (4.7, 5.5)	451.3 (410.4-491.2)	2.8 (2.6, 3.1)
	Caribbean	2003.3 (1825.4-2178.9)	13.3 (12.7, 13.9)	4010.5 (3175.3-4897.6)	16.4 (15.9, 16.9)	700.7 (622-793.4)	4.7 (4.3, 5)	1194.9 (910.4-1502.5)	4.9 (4.6, 5.2)
	Central Europe	10220.4 (9804.4-10641.4)	15.9 (15.6, 16.2)	11795.2 (9939.4-14053)	17.2 (16.9, 17.6)	3053.8 (2973-3136.4)	4.8 (4.6, 4.9)	2088.7 (1773.9-2451.5)	3 (2.9, 3.2)
	Eastern Europe	17469.4 (16717.9-18417.5)	15.8 (15.6, 16.1)	21674.3 (17985.3-26158.4)	17.6 (17.4, 17.9)	5338.2 (5140.1-5589)	4.9 (4.7, 5)	4333.2 (3601.4-5238.4)	3.5 (3.4, 3.6)
	Western Europe	52039.6 (50419.1-53777.5)	25.4 (25.1, 25.6)	59394.1 (50003.8-69938.6)	24.7 (24.5, 24.9)	11030.7 (10835-11242.2)	5.4 (5.3, 5.5)	7185.5 (6867.5-7509.9)	3 (2.9, 3)
	Andean Latin America	896.3 (788.8-1018.1)	6.2 (5.8, 6.6)	2954.3 (2246.2-3790.5)	9.2 (8.9, 9.6)	451.8 (401.4-513.4)	3.1 (2.9, 3.5)	843.3 (648.7-1083.5)	2.6 (2.5, 2.8)
	Central Latin America	4907.4 (4734-5086.4)	7.9 (7.7, 8.1)	16076.5 (13150.9-19567.4)	12.3 (12.1, 12.5)	1868.4 (1815.7-1920.1)	3 (2.9, 3.2)	3786.9 (3135.7-4558.6)	2.9 (2.8, 3)
	Southern Latin America	3060.1 (2860.6-3275.4)	13.2 (12.7, 13.6)	5524.5 (4019-7322.8)	15.3 (14.9, 15.8)	1232 (1179.3-1287.7)	5.3 (5, 5.6)	1381.4 (1264.8-1497.8)	3.8 (3.6, 4)
	Tropical Latin America	6303.1 (6045.5-6593.2)	9.8 (9.5, 10)	16561.4 (15390.9-17848.8)	13 (12.8, 13.2)	2559 (2464.2-2657.4)	4 (3.8, 4.1)	4356.7 (4078.1-4673.5)	3.4 (3.3, 3.5)
	North Africa and Middle East	8993.3 (8063.8-10137.6)	7.3 (7.2, 7.5)	43491.5 (36982-50548.7)	13.2 (13.1, 13.3)	4148.4 (3748.4-4739)	3.4 (3.3, 3.5)	11426.9 (9698-13499.3)	3.5 (3.4, 3.6)
	High-income North America	46972.1 (45578.5-48289.6)	30.6 (30.4, 30.9)	43402.7 (34889.3-53837.4)	23.1 (22.8, 23.3)	7915.9 (7750.9-8074.6)	5.2 (5.1, 5.3)	5359.9 (5078.5-5649.8)	2.8 (2.8, 2.9)
	Oceania	424 (323.3-543.6)	17 (15.4, 18.7)	1528.2 (1139.7-2022)	25.3 (24, 26.6)	239.1 (180.7-305.1)	9.7 (8.5, 11)	768.5 (572.4-1017.4)	12.8 (11.9, 13.7)
	Central Sub-Saharan Africa	1087.7 (813.4-1417.6)	6.1 (5.7, 6.4)	3773.1 (2690.8-5038.6)	7.8 (7.6, 8.1)	726.4 (558.8-928.3)	4.1 (3.8, 4.4)	2143.2 (1535.8-2881.2)	4.5 (4.3, 4.7)
	Eastern Sub-Saharan Africa	3163 (2485-3839.7)	5.3 (5.1, 5.5)	9718.2 (7943.7-11706.5)	6.5 (6.3, 6.6)	2127.5 (1661.3-2563.5)	3.6 (3.4, 3.8)	5252.4 (4293.3-6348.6)	3.5 (3.4, 3.6)
	Southern Sub-Saharan Africa	1797.7 (1585.5-2005.5)	8.9 (8.5, 9.4)	3524.6 (2943.3-4188.6)	9 (8.7, 9.3)	929.6 (824-1033.7)	4.7 (4.4, 5)	1540.9 (1281-1851.8)	3.9 (3.8, 4.2)
	Western Sub-Saharan Africa	3497.8 (2738.5-4452.2)	5.4 (5.2, 5.6)	13872.5 (10392.2-17974.7)	8.5 (8.4, 8.7)	2200.5 (1752.7-2826.1)	3.4 (3.3, 3.6)	7018.5 (5309.3-9219.2)	4.3 (4.2, 4.4)
	Global	106306.3 (100111.4-113467.8)	4.7 (4.6, 4.7)	136946.2 (123422.3-150290.4)	3.3 (3.3, 3.3)	91573.6 (85931-98019.2)	4 (4, 4)	110729.4 (101076.6-120624.3)	2.7 (2.7, 2.7)
**TBL**	Low SDI	2309.4 (1884.7-2835.5)	1.3 (1.3, 1.4)	5971.9 (5003.9-7231.2)	1.4 (1.4, 1.5)	2091.1 (1717.3-2574.8)	1.2 (1.2, 1.3)	5404.3 (4517.7-6571.7)	1.3 (1.3, 1.4)
**cancer**	Low-middle SDI	9127.7 (8200.1-10297.6)	2.1 (2.1, 2.2)	18936.2 (16744-21061.4)	2.2 (2.2, 2.2)	8234.6 (7345.2-9340.4)	1.9 (1.9, 2)	16773.6 (14930.5-18639.2)	2 (1.9, 2)
	Middle SDI	31522.5 (28464.1-34800.6)	4.4 (4.4, 4.5)	51333.3 (44548.7-58301)	3.7 (3.7, 3.8)	28262.2 (25418.4-31356.9)	4 (3.9, 4)	42952.5 (37467.1-48693.4)	3.1 (3.1, 3.1)

	High-middle SDI	36355 (33963.5-39300.2)	6.8 (6.7, 6.9)	39890.6 (35596.5-44418.8)	4.5 (4.5, 4.6)	31818.6 (29616.6-34315.3)	6 (5.9, 6)	31370.6 (28381.4-34559.9)	3.5 (3.5, 3.6)
	High SDI	26944.2 (26511.4-27353.1)	6 (6, 6.1)	20741.6 (18643.1-22901.8)	3.5 (3.5, 3.6)	21125.7 (20836.3-21426.2)	4.7 (4.7, 4.8)	14166.8 (13567.6-14792.6)	2.4 (2.4, 2.5)
	High-income Asia Pacific	3882.6 (3763.5-3989.8)	3.8 (3.7, 3.9)	3158.8 (2775.1-3594.9)	2.7 (2.6, 2.8)	2887.7 (2806.7-2958.9)	2.8 (2.7, 2.9)	1747.3 (1648.8-1849.1)	1.5 (1.4, 1.6)
	Central Asia	1853.6 (1778.7-1926.4)	7.6 (7.3, 8)	1671.7 (1479.1-1894.5)	3.5 (3.3, 3.7)	1632.9 (1564.9-1697.4)	6.8 (6.5, 7.2)	1456 (1290.1-1652.2)	3 (2.9, 3.2)
	East Asia	36487.8 (31228.2-42291.2)	6.5 (6.4, 6.5)	54862.4 (45569-65209.5)	5.8 (5.7, 5.8)	32568.6 (27591.2-37929.5)	5.8 (5.7, 5.9)	43343.3 (36305-51578.9)	4.5 (4.5, 4.6)
	South Asia	5726.7 (4881.5-6509.9)	1.3 (1.3, 1.4)	14532.6 (12383.6-16642.7)	1.6 (1.6, 1.7)	5199.6 (4484.3-5959.1)	1.2 (1.2, 1.2)	13004.3 (11016.6-14900.1)	1.5 (1.4, 1.5)
	Southeast Asia	6762.8 (5969.9-7603.6)	3.7 (3.6, 3.8)	13941 (11257-16662.3)	3.7 (3.6, 3.7)	6109.2 (5395.1-6841.4)	3.4 (3.3, 3.5)	12279.2 (9985.2-14491.7)	3.2 (3.2, 3.3)
	Australasia	479.8 (455.9-506.4)	4.4 (4, 4.8)	515.2 (400.3-660.7)	3.2 (2.9, 3.4)	351.4 (334.8-368.6)	3.2 (2.9, 3.6)	328.8 (297.4-362.7)	2 (1.8, 2.2)
	Caribbean	585 (544.1-631.5)	3.9 (3.6, 4.3)	709.3 (568-867.6)	2.9 (2.7, 3.1)	514.6 (477.9-556.6)	3.5 (3.2, 3.8)	602.5 (482.6-734)	2.4 (2.2, 2.6)
	Central Europe	5959.9 (5816.7-6108)	9.5 (9.2, 9.7)	3819 (3280.9-4405.5)	5.5 (5.3, 5.6)	5271.3 (5150.4-5400)	8.4 (8.1, 8.6)	3171.8 (2705-3654.7)	4.5 (4.4, 4.7)
	Eastern Europe	8674.1 (8154.6-9032.5)	8.2 (8.1, 8.4)	5908.5 (5175.6-6758.2)	4.8 (4.7, 4.9)	7429.8 (6991.9-7725.6)	7.1 (6.9, 7.2)	4696.6 (4047.4-5353.6)	3.8 (3.7, 3.9)
	Western Europe	12812.8 (12545.6-13102.5)	6.2 (6.1, 6.3)	10578.7 (8724.1-12465.2)	4.2 (4.1, 4.3)	10396.6 (10201.3-10604.7)	5 (4.9, 5.1)	7431.9 (6968.6-7817.3)	2.9 (2.9, 3)
	Andean Latin America	362.3 (311-412.3)	2.4 (2.2, 2.7)	602.7 (456.3-788.3)	1.9 (1.7, 2)	320.1 (274.4-365.3)	2.2 (2, 2.4)	515.5 (389.2-671.9)	1.6 (1.5, 1.8)
	Central Latin America	1418 (1384.5-1453.9)	2.3 (2.1, 2.4)	2227.5 (1841.5-2671.2)	1.7 (1.6, 1.8)	1248.5 (1218.6-1282.3)	2 (1.9, 2.1)	1877.9 (1553.9-2268)	1.4 (1.4, 1.5)
	Southern Latin America	1449.1 (1379.8-1523.3)	6.3 (6, 6.6)	1095.4 (817.6-1443)	3 (2.9, 3.2)	1301.9 (1238.5-1365.4)	5.6 (5.3, 6)	921.7 (830.4-1031.6)	2.5 (2.4, 2.7)
	Tropical Latin America	1901.2 (1841.3-1968)	3 (2.9, 3.1)	2708.3 (2554.6-2865.1)	2.1 (2.1, 2.2)	1705.9 (1648.1-1766.6)	2.7 (2.6, 2.8)	2353.6 (2222.1-2485.8)	1.9 (1.8, 1.9)
	North Africa and Middle East	3777.1 (2962.8-4633.1)	3.1 (3, 3.2)	8046.6 (7010-9194.7)	2.5 (2.4, 2.5)	3425.3 (2674.6-4212.9)	2.9 (2.8, 3)	7098.2 (6158.6-8155.7)	2.2 (2.1, 2.3)
	High-income North America	11532.9 (11314.8-11759.1)	7.7 (7.6, 7.9)	7013.9 (6002.9-8224.9)	3.6 (3.5, 3.7)	8825.3 (8662.3-8993.5)	5.9 (5.8, 6.1)	4878.8 (4673.9-5083.4)	2.5 (2.4, 2.6)
	Oceania	92.3 (68.9-134.6)	3.7 (3, 4.6)	244.4 (171.1-362)	4 (3.5, 4.6)	81.5 (60.9-118.8)	3.3 (2.6, 4.1)	216.8 (150.7-322)	3.6 (3.1, 4.1)
	Central Sub-Saharan Africa	421.2 (237.3-848.8)	2.5 (2.3, 2.7)	974.6 (606.3-1753.6)	2.1 (2, 2.3)	379.6 (213.8-762.5)	2.3 (2, 2.5)	885.1 (548.8-1601.6)	1.9 (1.8, 2.1)
	Eastern Sub-Saharan Africa	566.9 (466-710.4)	1 (0.9, 1.1)	1425.7 (1126.1-1806.8)	1 (0.9, 1)	511.3 (417.7-640.4)	0.9 (0.8, 1)	1304.6 (1034-1639.8)	0.9 (0.9, 1)
	Southern Sub-Saharan Africa	903.1 (805.3-1029.7)	4.8 (4.5, 5.2)	1074 (929.2-1231.7)	2.8 (2.7, 3)	823.3 (729.1-934.6)	4.4 (4.1, 4.7)	967 (841.2-1105.6)	2.6 (2.4, 2.7)
	Western Sub-Saharan Africa	657 (529.4-796.5)	1 (0.9, 1.1)	1835.8 (1459.4-2241.7)	1.1 (1.1, 1.2)	589.4 (480.4-715.8)	0.9 (0.8, 1)	1648.4 (1312.8-2024.7)	1 (1, 1.1)
**CRC**	Global	94707.1 (90420.5-99416.4)	4 (4, 4)	225736 (207658-246755.7)	5.5 (5.5, 5.5)	50436.5 (47475-53367.8)	2.1 (2.1, 2.2)	86545.6 (80162-93431.1)	2.1 (2.1, 2.1)
	Low SDI	2777 (2284.1-3327.5)	1.5 (1.5, 1.6)	7716.4 (6643-8911.9)	1.8 (1.8, 1.8)	2225.6 (1836.4-2671)	1.2 (1.2, 1.3)	5693 (4933-6582.5)	1.3 (1.3, 1.4)

Low-middle SDI	8615 (7710.5-9669.5)	1.9 (1.9, 2)	24979.5 (22488.3-27829.7)	2.9 (2.8, 2.9)	6433.1 (5715.1-7231.1)	1.5 (1.4, 1.5)	15376.2 (13666.2-17136.9)	1.8 (1.7, 1.8)
Middle SDI	23377.9 (21458.4-25471.2)	3.1 (3.1, 3.1)	75841.7 (67256.8-85383.6)	5.6 (5.5, 5.6)	15311.1 (13878-16640)	2.1 (2, 2.1)	31223.5 (28190.6-34399.3)	2.3 (2.3, 2.3)
High-middle SDI	27712.9 (26289.2-29502.6)	5 (4.9, 5.1)	69605.6 (62366.1-77641.5)	8.1 (8, 8.1)	14897.7 (14014.1-15826.3)	2.7 (2.7, 2.7)	22183.4 (20248.8-24368.7)	2.6 (2.5, 2.6)
High SDI	32179.4 (31645.5-32734.2)	7.2 (7.1, 7.3)	47489.7 (43601.5-51742.5)	8.4 (8.3, 8.5)	11544 (11365.8-11718.1)	2.6 (2.5, 2.6)	12020.1 (11559.8-12508.9)	2.1 (2.1, 2.1)
High-income Asia Pacific	7952.7 (7713.7-8186.5)	7.8 (7.6, 8)	9294.5 (8006.7-10617.2)	8.3 (8.1, 8.5)	2837.1 (2778.7-2891.7)	2.8 (2.7, 2.9)	2056.4 (1941.2-2159.5)	1.8 (1.7, 1.9)
Central Asia	1297.1 (1243.5-1358.5)	4.8 (4.5, 5.1)	1830.9 (1636.7-2062.3)	3.8 (3.6, 4)	805.5 (773.3-844)	3 (2.8, 3.3)	982.6 (878.4-1113.7)	2 (1.9, 2.2)
East Asia	25347.7 (22184.8-28937.6)	4.2 (4.2, 4.3)	90911 (76318.1-106893.9)	10 (10, 10.1)	15532.2 (13405-17867.5)	2.6 (2.6, 2.7)	27447.4 (23104-32222.8)	3 (3, 3)
South Asia	6135.9 (5396.9-6948.6)	1.4 (1.3, 1.4)	18253.4 (15671.1-21008.5)	2 (2, 2.1)	4824.3 (4265.5-5454.8)	1.1 (1.1, 1.1)	12420.9 (10757.6-14391.2)	1.4 (1.4, 1.4)
Southeast Asia	6101.4 (5194.6-6824)	3.2 (3.1, 3.2)	18976.3 (15675-22334.4)	5 (5, 5.1)	4241 (3616.7-4793)	2.2 (2.2, 2.3)	10550.2 (8800.3-12385.5)	2.8 (2.7, 2.8)
Australasia	941.4 (892.5-992.7)	8.6 (8, 9.2)	1520.4 (1194.1-1932.8)	9.7 (9.2, 10.2)	320.6 (306.9-335.5)	2.9 (2.6, 3.3)	337.1 (308-369.5)	2.1 (1.9, 2.4)
Caribbean	575.4 (540.2-611.4)	3.7 (3.4, 4)	1222 (1005.9-1474)	5 (4.7, 5.3)	314.2 (292.3-338.6)	2 (1.8, 2.3)	561.9 (461.2-681.1)	2.3 (2.1, 2.5)
Central Europe	3846.7 (3736.7-3969.4)	6.1 (5.9, 6.3)	4850.4 (4151.5-5578.9)	7.2 (7, 7.4)	2112.8 (2056.6-2174.4)	3.3 (3.2, 3.5)	1861.5 (1598.2-2134)	2.7 (2.6, 2.9)
Eastern Europe	6810.7 (6347.7-7171.6)	6.2 (6.1, 6.4)	9238.7 (8187.5-10479.7)	7.6 (7.5, 7.8)	3514.6 (3276-3697.7)	3.2 (3.1, 3.3)	3458.8 (3063-3897.6)	2.8 (2.8, 2.9)
Western Europe	13441.6 (13128.9-13761.4)	6.6 (6.5, 6.7)	17020.9 (14619.3-19670.2)	7.1 (7, 7.2)	5082.1 (4991.8-5173.5)	2.5 (2.4, 2.5)	4247 (4053.5-4426.3)	1.8 (1.7, 1.8)
Andean Latin America	351.1 (310.6-399.2)	2.3 (2.1, 2.6)	1507.4 (1159.9-1950.9)	4.7 (4.4, 4.9)	203.3 (181.1-230.3)	1.4 (1.2, 1.6)	525.7 (410.9-669.4)	1.6 (1.5, 1.8)
Central Latin America	1452.3 (1412.9-1490.4)	2.2 (2.1, 2.3)	5782.2 (4916.4-6815)	4.4 (4.3, 4.5)	875.6 (852.8-898)	1.4 (1.3, 1.5)	2564 (2170.9-3008.6)	2 (1.9, 2)
Southern Latin America	949.7 (903.3-999.4)	4.1 (3.8, 4.3)	2233.3 (1694.2-2912.9)	6.2 (6, 6.5)	584.4 (558-613.2)	2.5 (2.3, 2.7)	1021.5 (936.1-1116.3)	2.8 (2.7, 3)
Tropical Latin America	1916.8 (1856.5-1983.1)	2.9 (2.7, 3)	5754.9 (5414.7-6061.3)	4.6 (4.4, 4.7)	1239 (1200.6-1282.7)	1.9 (1.8, 2)	2856.8 (2697.7-3003.1)	2.3 (2.2, 2.3)
North Africa and Middle East	3125.8 (2608.2-3801.4)	2.5 (2.4, 2.5)	11101.1 (9615.5-12783.3)	3.4 (3.3, 3.4)	2179.8 (1816-2650.2)	1.7 (1.7, 1.8)	5485 (4715.8-6362.1)	1.7 (1.6, 1.7)
High-income North America	11664.8 (11348.3-11962.4)	7.7 (7.5, 7.8)	18499.3 (15902.1-21546.9)	9.8 (9.7, 10)	3604.5 (3516.8-3688.1)	2.4 (2.3, 2.5)	4544.6 (4398.5-4709)	2.4 (2.3, 2.5)
Oceania	61 (48.7-76)	2.4 (1.8, 3.1)	164.6 (125.9-215.1)	2.7 (2.3, 3.1)	42.8 (33.7-53.6)	1.7 (1.2, 2.3)	111.8 (84.6-147.5)	1.8 (1.5, 2.2)
Central Sub-Saharan Africa	291.3 (215.4-386.8)	1.6 (1.4, 1.8)	797.7 (566.4-1081.9)	1.6 (1.5, 1.8)	232 (177.1-305.3)	1.3 (1.1, 1.5)	604.2 (427.9-820.8)	1.3 (1.2, 1.4)
Eastern Sub-Saharan Africa	1066.6 (858.1-1310.7)	1.8 (1.6, 1.9)	3089 (2525.8-3770.4)	2 (2, 2.1)	849.7 (678.3-1042.6)	1.4 (1.3, 1.5)	2298.2 (1884.8-2807.8)	1.5 (1.5, 1.6)
Southern Sub-Saharan Africa	565.9 (507.9-631.5)	2.8 (2.6, 3)	1111.1 (955.2-1276.6)	2.8 (2.6, 3)	405.9 (366.2-451.8)	2 (1.8, 2.2)	738 (635-851.9)	1.9 (1.7, 2)
Western Sub-Saharan Africa	811.1 (642.4-1008.6)	1.2 (1.1, 1.3)	2577 (2059.5-3111.6)	1.5 (1.5, 1.6)	635.1 (500.1-791.2)	1 (0.9, 1)	1872 (1493.9-2315.5)	1.1 (1.1, 1.2)

**Stomach cancer**	Global	125974.3 (118139.9-133955.5)	5.4 (5.3, 5.4)	144321.2 (130472.9-158788.9)	3.5 (3.5, 3.6)	97382.7 (90808.2-104061.7)	4.2 (4.1, 4.2)	87333.5 (79904.3-95000.1)	2.1 (2.1, 2.1)
	Low SDI	5835.5 (4920.5-6622.3)	3.2 (3.1, 3.3)	9441.7 (8062-10865.2)	2.2 (2.2, 2.3)	5185.9 (4411-5845.1)	2.9 (2.8, 3)	8351.3 (7187-9592)	2 (1.9, 2)
	Low-middle SDI	18731.3 (16680.3-20403.8)	4.2 (4.2, 4.3)	26153 (23536-28918.2)	3 (3, 3)	16458.5 (14722.8-17964.3)	3.8 (3.7, 3.8)	21408.6 (19236-23771.6)	2.5 (2.4, 2.5)
	Middle SDI	41707 (37519.7-46081.8)	5.7 (5.7, 5.8)	54281.9 (47258.6-61911.5)	4 (3.9, 4)	34945.5 (31453.6-38870.9)	4.8 (4.8, 4.9)	31097.3 (27804.1-34949.7)	2.3 (2.2, 2.3)
	High-middle SDI	35029.6 (32514.5-37646.2)	6.4 (6.3, 6.5)	40349.1 (35304.3-45650.3)	4.7 (4.6, 4.7)	27950.4 (25910.2-30134)	5.1 (5.1, 5.2)	20715.7 (18484-22953.5)	2.4 (2.3, 2.4)
	High SDI	24633 (24088.8-25166.5)	5.5 (5.4, 5.6)	14041.5 (12929.2-15275.4)	2.5 (2.5, 2.5)	12811 (12569-13027.3)	2.9 (2.8, 2.9)	5717.8 (5462.9-5995.2)	1 (1, 1)
	High-income Asia Pacific	16694.8 (16188.1-17189.8)	16.5 (16.3, 16.8)	6407.9 (5627.8-7282.8)	5.9 (5.8, 6.1)	7715.8 (7482.3-7894.4)	7.7 (7.5, 7.8)	2069 (1943.4-2212.1)	1.9 (1.8, 2)
	Central Asia	2161.1 (2076.7-2244.2)	8.5 (8.1, 8.8)	1974.4 (1764.8-2229.9)	4.1 (3.9, 4.3)	1875 (1799-1947.7)	7.5 (7.1, 7.8)	1679.7 (1497.9-1900)	3.5 (3.3, 3.7)
	East Asia	49304.8 (42924.4-56327.8)	8.6 (8.6, 8.7)	68382.2 (56611.1-81594.8)	7.4 (7.4, 7.5)	40658 (34888.9-46565.6)	7.2 (7.1, 7.3)	32211.4 (26787.3-38306)	3.4 (3.4, 3.5)
	South Asia	14580.2 (12911.5-16083.5)	3.2 (3.2, 3.3)	21127.2 (18261.3-24233.1)	2.3 (2.3, 2.4)	12915.2 (11397-14184.9)	2.9 (2.8, 2.9)	18314.2 (15927.6-21109.5)	2 (2, 2.1)
	Southeast Asia	5693.3 (4809.9-6436.8)	3 (2.9, 3.1)	6354.4 (5397.3-7406.5)	1.7 (1.6, 1.7)	4947.9 (4220.1-5516.7)	2.6 (2.5, 2.7)	5019.6 (4323.5-5812.6)	1.3 (1.3, 1.4)
	Australasia	210.7 (198.4-225.6)	1.9 (1.7, 2.2)	241.8 (185.8-310.1)	1.5 (1.4, 1.8)	118.3 (112.2-124.7)	1.1 (0.9, 1.3)	102.1 (90.4-115.2)	0.6 (0.5, 0.8)
	Caribbean	409.5 (348.7-452.6)	2.7 (2.4, 2.9)	534.8 (432.1-643.9)	2.2 (2, 2.4)	346.8 (292.9-385.5)	2.3 (2, 2.5)	436.6 (348.7-528.9)	1.8 (1.6, 2)
	Central Europe	2511.2 (2452.4-2570.1)	3.9 (3.8, 4.1)	1460.4 (1262.4-1678.1)	2.2 (2, 2.3)	2128.3 (2076.8-2181)	3.3 (3.2, 3.5)	1086.3 (933.4-1254.5)	1.6 (1.5, 1.7)
	Eastern Europe	9947.9 (9280.4-10297.7)	9.1 (8.9, 9.3)	6053.2 (5378.7-6793.4)	5 (4.9, 5.1)	7650.9 (7151.3-7922.7)	7 (6.9, 7.2)	3706.9 (3262.8-4180.3)	3 (2.9, 3.1)
	Western Europe	6142.6 (6007.9-6269.8)	3 (2.9, 3.1)	4611.8 (3923.7-5399)	1.9 (1.9, 2)	4002.3 (3924.6-4072)	2 (1.9, 2)	2168.7 (2043-2296.1)	0.9 (0.9, 0.9)
	Andean Latin America	985.2 (889-1095.8)	6.5 (6.1, 7)	1510.6 (1177.3-1917.6)	4.7 (4.5, 4.9)	857.4 (773.2-951)	5.8 (5.4, 6.2)	1185.4 (924.7-1503.6)	3.7 (3.5, 3.9)
	Central Latin America	2590.3 (2514.5-2662.7)	4 (3.9, 4.2)	4706.9 (3923.5-5598.5)	3.6 (3.5, 3.7)	2136.9 (2072.6-2197.9)	3.4 (3.2, 3.5)	3253.4 (2730.4-3876.7)	2.5 (2.4, 2.6)
	Southern Latin America	845.1 (809.6-885.2)	3.6 (3.4, 3.9)	916 (704.2-1196.6)	2.6 (2.4, 2.7)	694.9 (666.7-726.7)	3 (2.8, 3.2)	642.9 (591.4-700.2)	1.8 (1.6, 1.9)
	Tropical Latin America	2493.3 (2410-2578.5)	3.8 (3.7, 4)	3064.3 (2911-3226.3)	2.4 (2.3, 2.5)	2154.1 (2077.5-2231)	3.3 (3.2, 3.5)	2408.9 (2287.8-2525.5)	1.9 (1.8, 2)
	North Africa and Middle East	4441 (3842.7-4988.3)	3.5 (3.4, 3.6)	7272.1 (6170.9-8484)	2.2 (2.2, 2.3)	3858.9 (3342.9-4340.6)	3.1 (3, 3.2)	5676.5 (4740.1-6694.7)	1.7 (1.7, 1.8)
	High-income North America	2492.6 (2423.1-2554.1)	1.6 (1.6, 1.7)	2785.3 (2404.8-3259.4)	1.5 (1.4, 1.6)	1370.1 (1335.5-1400.6)	0.9 (0.9, 0.9)	1226.3 (1174.3-1282.5)	0.7 (0.6, 0.7)
	Oceania	126.4 (95.4-158.4)	4.9 (4.1, 5.9)	293.5 (212-392.5)	4.8 (4.2, 5.3)	108.7 (82.1-136.5)	4.3 (3.5, 5.2)	252.2 (182.5-337.9)	4.1 (3.6, 4.7)
	Central Sub-Saharan Africa	544.3 (412-697.8)	3 (2.8, 3.3)	905.5 (672.9-1187.3)	1.9 (1.8, 2)	480.4 (369.3-599)	2.7 (2.5, 3)	805.2 (598.7-1067.4)	1.7 (1.6, 1.8)

Eastern Sub-Saharan Africa	1916.8 (1532.4-2213.4)	3.2 (3, 3.3)	2711.2 (2256.9-3264.7)	1.8 (1.7, 1.9)	1694.9 (1349.6-1954)	2.8 (2.7, 3)	2432.5 (2017.1-2917)	1.6 (1.6, 1.7)
Southern Sub-Saharan Africa	562.1 (514.3-608.5)	2.8 (2.6, 3.1)	618.6 (522.2-727.8)	1.6 (1.5, 1.7)	492.3 (450.7-533.2)	2.5 (2.3, 2.7)	535 (454.6-630.1)	1.4 (1.3, 1.5)
Western Sub-Saharan Africa	1320.8 (1108.7-1516.4)	2 (1.9, 2.1)	2389.2 (1933.4-2896)	1.4 (1.4, 1.5)	1175.6 (999.6-1359.9)	1.8 (1.7, 1.9)	2120.4 (1707.2-2570)	1.3 (1.2, 1.3)

Abbreviations: ASR, age-standardized rate; TBL, racheal, bronchus, and lung; CRC, colorectal cancer.

Table S7. Incidence and and death of all early-onset cancers in 1990 and 2019 by region and sociodemographic index level.

Location	Incidence		Death		DALYs	
	1990 ASR per 100k	2019 ASR per 100k	1990 ASR per 100k	2019 ASR per 100k	1990 ASR per 100k	2019 ASR per 100k
Global	75.7 (75.6, 75.8)	80.3 (80.2, 80.4)	34.7 (34.6, 34.8)	26.1 (26.1, 26.2)	1727.9 (1727.3, 1728.4)	1321.7 (1321.4, 1322.1)
Low SDI	43.2 (42.9, 43.5)	45.3 (45.1, 45.5)	28.4 (28.2, 28.7)	26.8 (26.6, 26.9)	1409 (1407.3, 1410.8)	1334.7 (1333.7, 1335.8)
Low-middle SDI	45.5 (45.3, 45.7)	52.8 (52.6, 52.9)	29.5 (29.3, 29.6)	27.7 (27.6, 27.8)	1473.8 (1472.7, 1475)	1392.9 (1392.1, 1393.7)
Middle SDI	59 (58.8, 59.2)	68.9 (68.8, 69.1)	37 (36.9, 37.2)	26.8 (26.7, 26.9)	1841.2 (1840.2, 1842.2)	1356.1 (1355.5, 1356.7)
High-middle SDI	81.5 (81.3, 81.7)	94.5 (94.3, 94.7)	40.5 (40.3, 40.7)	26.9 (26.8, 27)	2007.1 (2006, 2008.3)	1368.5 (1367.7, 1369.3)
High SDI	139.1 (138.8, 139.5)	159.7 (159.4, 160)	31.2 (31, 31.3)	19.8 (19.7, 20)	1563.2 (1562, 1564.3)	1023 (1022.1, 1023.8)
High-income Asia Pacific	82.2 (81.6, 82.8)	86.1 (85.5, 86.7)	29.5 (29.2, 29.9)	16.3 (16, 16.5)	1486.6 (1484.2, 1489)	840.6 (838.7, 842.4)
Central Asia	78.8 (77.7, 79.9)	72.5 (71.7, 73.2)	42.3 (41.5, 43.2)	31.8 (31.3, 32.3)	2115.5 (2109.9, 2121.2)	1609.7 (1606.1, 1613.3)
East Asia	69.1 (68.9, 69.3)	92.5 (92.3, 92.7)	46.6 (46.4, 46.8)	29.6 (29.5, 29.7)	2301.7 (2300.5, 2302.9)	1502.2 (1501.3, 1503)
South Asia	38.3 (38.1, 38.5)	46.3 (46.1, 46.4)	25.6 (25.4, 25.7)	25.9 (25.8, 26)	1281.6 (1280.5, 1282.6)	1301.7 (1300.9, 1302.4)
Southeast Asia	53.8 (53.5, 54.1)	61.1 (60.8, 61.3)	32.5 (32.3, 32.8)	28.4 (28.3, 28.6)	1631.2 (1629.4, 1633)	1437.3 (1436, 1438.5)
Australasia	146.2 (144, 148.5)	157.7 (155.7, 159.7)	28.6 (27.6, 29.6)	20 (19.3, 20.7)	1456.1 (1448.9, 1463.2)	1044.3 (1039.1, 1049.5)
Caribbean	69.3 (68, 70.6)	75.3 (74.3, 76.4)	32.4 (31.5, 33.3)	30.3 (29.6, 31)	1626.8 (1620.5, 1633.1)	1529.7 (1524.8, 1534.6)
Central Europe	98.3 (97.5, 99.1)	103.7 (102.9, 104.5)	44.8 (44.3, 45.3)	29.8 (29.4, 30.2)	2201.6 (2197.9, 2205.3)	1484.7 (1481.7, 1487.8)
Eastern Europe	93.5 (92.9, 94.1)	108.8 (108.1, 109.4)	43.4 (43, 43.8)	33.7 (33.4, 34)	2146.1 (2143.3, 2148.9)	1698.6 (1696.2, 1701)
Western Europe	118.1 (117.6, 118.6)	125.6 (125.1, 126.1)	32.6 (32.3, 32.8)	20.4 (20.2, 20.6)	1633.7 (1631.9, 1635.4)	1057.2 (1055.8, 1058.6)
Andean Latin America	57.9 (56.7, 59.1)	68.8 (67.9, 69.7)	32.1 (31.2, 33)	26.2 (25.6, 26.8)	1620.7 (1614.4, 1627.1)	1349.9 (1345.9, 1353.9)

Central Latin America	60.8 (60.2, 61.4)	72.7 (72.3, 73.2)	27.7 (27.3, 28.1)	24.2 (23.9, 24.5)	1405.2 (1402.3, 1408.1)	1249.9 (1248, 1251.8)
Southern Latin America	86.8 (85.6, 88)	94.5 (93.5, 95.5)	39.8 (39, 40.6)	28.5 (27.9, 29)	1977.4 (1971.7, 1983.1)	1452.6 (1448.6, 1456.5)
Tropical Latin America	75.8 (75.1, 76.5)	83.8 (83.3, 84.3)	31.9 (31.4, 32.3)	26.1 (25.8, 26.3)	1579.9 (1576.9, 1582.9)	1316.8 (1314.8, 1318.8)
North Africa and Middle East	44.8 (44.4, 45.1)	58.9 (58.7, 59.2)	26.4 (26.2, 26.7)	22.2 (22.1, 22.4)	1325.4 (1323.4, 1327.3)	1128.8 (1127.6, 1129.9)
High-income North America	215.6 (214.8, 216.3)	273.2 (272.5, 274)	30.7 (30.4, 31)	20.3 (20.1, 20.5)	1551.6 (1549.6, 1553.6)	1048.1 (1046.6, 1049.6)
Oceania	59.8 (56.8, 62.9)	69.6 (67.5, 71.7)	36.4 (34.1, 38.9)	39.1 (37.6, 40.7)	1814.7 (1798.2, 1831.3)	1952.6 (1941.6, 1963.7)
Central Sub-Saharan Africa	45 (44, 46)	42.5 (41.9, 43.1)	29.2 (28.4, 30)	25.5 (25, 25.9)	1430.1 (1424.6, 1435.6)	1253.4 (1250.3, 1256.6)
Eastern Sub-Saharan Africa	49.7 (49.2, 50.3)	46.9 (46.6, 47.3)	31.9 (31.5, 32.4)	27.6 (27.3, 27.8)	1578.6 (1575.5, 1581.8)	1373.1 (1371.2, 1374.9)
Southern Sub-Saharan Africa	70.3 (69.2, 71.5)	63.3 (62.5, 64.1)	38.9 (38, 39.8)	30.4 (29.9, 31)	1919.4 (1913.3, 1925.5)	1506.9 (1503.1, 1510.7)
Western Sub-Saharan Africa	33.4 (33, 33.8)	37.4 (37.1, 37.7)	20.9 (20.5, 21.2)	21.2 (21, 21.4)	1033.1 (1030.6, 1035.5)	1052.5 (1050.9, 1054)

Abbreviations: ASR, age-standardized rate.

Figure S1

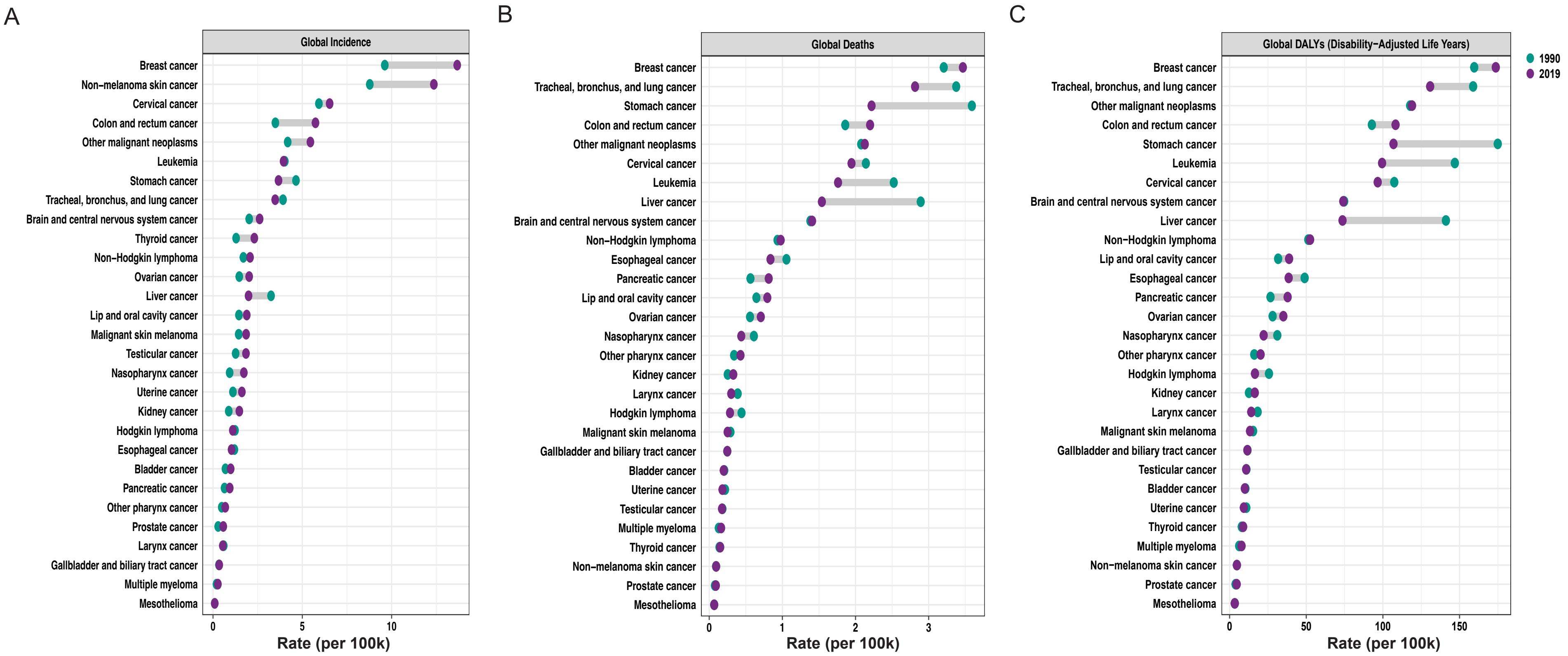
A
Global Incidence
Breast cancer
Non−melanoma skin cancer
Cervical cancer
Colon and rectum cancer
Other malignant neoplasms
Leukemia
Stomach cancer
Tracheal, bronchus, and lung cancer
Brain and central nervous system cancer
Thyroid cancer
Non−Hodgkin lymphoma
Ovarian cancer
Liver cancer
Lip and oral cavity cancer
Malignant skin melanoma
Testicular cancer
Nasopharynx cancer
Uterine cancer
Kidney cancer
Hodgkin lymphoma
Esophageal cancer
Bladder cancer
Pancreatic cancer
Other pharynx cancer
Prostate cancer
Larynx cancer
Gallbladder and biliary tract cancer
Multiple myeloma
Mesothelioma
0
5
10
Rate (per 100k)
B
Global Deaths
Breast cancer
Tracheal, bronchus, and lung cancer
Stomach cancer
Colon and rectum cancer
Other malignant neoplasms
Cervical cancer
Leukemia
Liver cancer
Brain and central nervous system cancer
Non−Hodgkin lymphoma
Esophageal cancer
Pancreatic cancer
Lip and oral cavity cancer
Ovarian cancer
Nasopharynx cancer
Other pharynx cancer
Kidney cancer
Larynx cancer
Hodgkin lymphoma
Malignant skin melanoma
Gallbladder and biliary tract cancer
Bladder cancer
Uterine cancer
Testicular cancer
Multiple myeloma
Thyroid cancer
Non−melanoma skin cancer
Prostate cancer
Mesothelioma
0
1
2
3
Rate (per 100k)
C
Global DALYs (Disability−Adjusted Life Years)
Breast cancer
Tracheal, bronchus, and lung cancer
Other malignant neoplasms
Colon and rectum cancer
Stomach cancer
Leukemia
Cervical cancer
Brain and central nervous system cancer
Liver cancer
Non−Hodgkin lymphoma
Lip and oral cavity cancer
Esophageal cancer
Pancreatic cancer
Ovarian cancer
Nasopharynx cancer
Other pharynx cancer
Hodgkin lymphoma
Kidney cancer
Larynx cancer
Malignant skin melanoma
Gallbladder and biliary tract cancer
Testicular cancer
Bladder cancer
Uterine cancer
Thyroid cancer
Multiple myeloma
Non−melanoma skin cancer
Prostate cancer
Mesothelioma
0
50
100
150
Rate (per 100k)
1990
2019

Figure S2

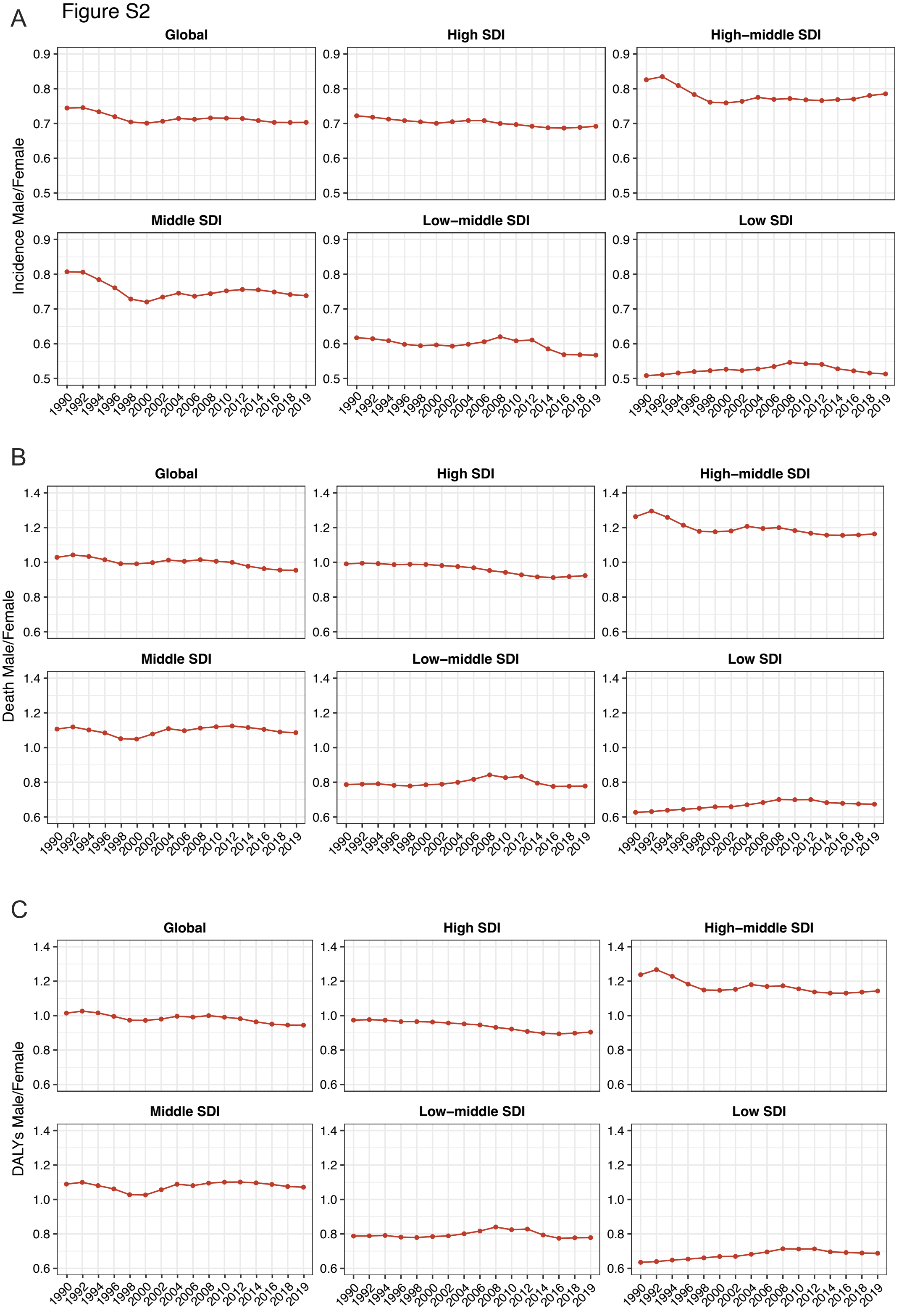
A
Global
High SDI
High−middle SDI
Middle SDI
Low−middle SDI
Low SDI
Incidence Male/Female
B
Death Male/Female
C
DALYs Male/Female

Figure S3

	Kidney cancer	Malignant.skin melanoma	Gallbladder and biliary tract cancer	Testicular cancer	Thyroid cancer	Prostate cancer	Mesothelioma	Non.melanoma skin cancer	Uterine cancer	Bladder cancer	Multiple myeloma	Other pharynx cancer	Nasopharynx cancer	Hodgkin lymphoma	Larynx cancer	Non Hodgkin lymphoma	Ovarian cancer	Pancreatic cancer	Esophageal cancer	Lip and oral cavity cancer	Brain and central nervous system cancer	Liver cancer	Colon and rectum cancer	Tracheal bronchus and lung cancer	Other malignant neoplasms	Leukemia	Stomach cancer	Breast cancer	Cervical cancer
Global	0.01	0.01	0.01	0.01	0.01	0.00	0.00	0.00	0.01	0.01	0.01	0.02	0.02	0.01	0.01	0.04	0.03	0.03	0.03	0.03	0.05	0.06	0.08	0.10	0.08	0.07	0.08	0.13	0.07
Low SDI	0.01	0.01	0.01	0.01	0.01	0.00	0.00	0.00	0.01	0.01	0.01	0.02	0.02	0.02	0.01	0.04	0.02	0.01	0.04	0.04	0.04	0.03	0.05	0.05	0.12	0.06	0.07	0.15	0.15
Low−middle SDI	0.01	0.00	0.01	0.01	0.01	0.00	0.00	0.00	0.01	0.01	0.01	0.03	0.02	0.01	0.02	0.04	0.03	0.02	0.03	0.05	0.04	0.04	0.06	0.07	0.10	0.06	0.09	0.15	0.09
Middle SDI	0.01	0.00	0.01	0.01	0.00	0.00	0.00	0.00	0.01	0.01	0.01	0.01	0.02	0.01	0.01	0.04	0.02	0.03	0.03	0.02	0.05	0.09	0.09	0.12	0.07	0.07	0.09	0.12	0.06
High−middle SDI	0.02	0.01	0.01	0.01	0.00	0.00	0.00	0.00	0.01	0.01	0.01	0.01	0.02	0.01	0.01	0.03	0.03	0.04	0.03	0.02	0.06	0.06	0.10	0.14	0.06	0.06	0.09	0.11	0.05
High SDI	0.02	0.03	0.01	0.01	0.00	0.00	0.00	0.00	0.01	0.01	0.01	0.01	0.01	0.01	0.01	0.04	0.03	0.05	0.03	0.02	0.07	0.04	0.11	0.13	0.07	0.06	0.05	0.14	0.04
High−income Asia Pacific	0.02	0.00	0.02	0.00	0.00	0.00	0.00	0.00	0.01	0.01	0.01	0.01	0.01	0.00	0.00	0.03	0.04	0.05	0.02	0.01	0.04	0.09	0.12	0.10	0.06	0.06	0.12	0.13	0.05
Central Asia	0.02	0.01	0.00	0.01	0.00	0.00	0.00	0.00	0.01	0.01	0.00	0.01	0.01	0.01	0.01	0.03	0.03	0.03	0.03	0.02	0.09	0.05	0.06	0.10	0.08	0.07	0.11	0.12	0.07
East Asia	0.01	0.00	0.01	0.00	0.00	0.00	0.00	0.00	0.01	0.01	0.01	0.00	0.02	0.00	0.00	0.03	0.02	0.04	0.05	0.01	0.05	0.13	0.10	0.16	0.04	0.07	0.12	0.06	0.04
South Asia	0.01	0.00	0.01	0.01	0.01	0.00	0.00	0.00	0.00	0.01	0.01	0.05	0.02	0.02	0.02	0.04	0.03	0.02	0.03	0.08	0.04	0.02	0.05	0.06	0.12	0.05	0.08	0.15	0.07
Southeast Asia	0.01	0.00	0.01	0.00	0.01	0.00	0.00	0.00	0.01	0.00	0.00	0.01	0.03	0.01	0.01	0.04	0.04	0.02	0.02	0.02	0.04	0.05	0.10	0.11	0.08	0.08	0.05	0.20	0.06
Australasia	0.02	0.07	0.01	0.01	0.00	0.00	0.01	0.01	0.01	0.01	0.01	0.01	0.01	0.01	0.00	0.04	0.03	0.04	0.02	0.02	0.09	0.03	0.11	0.11	0.07	0.05	0.03	0.15	0.03
Caribbean	0.02	0.01	0.01	0.00	0.01	0.01	0.00	0.01	0.02	0.01	0.01	0.01	0.01	0.01	0.01	0.05	0.02	0.02	0.02	0.01	0.04	0.02	0.08	0.08	0.07	0.07	0.06	0.16	0.15
Central Europe	0.02	0.03	0.01	0.01	0.00	0.00	0.00	0.00	0.01	0.01	0.01	0.02	0.01	0.01	0.02	0.03	0.04	0.05	0.02	0.03	0.08	0.02	0.09	0.16	0.06	0.05	0.05	0.11	0.06
Eastern Europe	0.03	0.03	0.00	0.01	0.00	0.00	0.00	0.00	0.01	0.01	0.01	0.02	0.00	0.02	0.01	0.03	0.04	0.05	0.02	0.03	0.07	0.02	0.09	0.12	0.06	0.05	0.09	0.11	0.06
Western Europe	0.02	0.04	0.01	0.01	0.00	0.00	0.00	0.00	0.01	0.01	0.01	0.01	0.00	0.01	0.01	0.04	0.03	0.05	0.02	0.02	0.09	0.03	0.09	0.15	0.07	0.05	0.05	0.15	0.03
Andean Latin America	0.02	0.01	0.02	0.02	0.01	0.01	0.00	0.01	0.01	0.00	0.01	0.00	0.00	0.01	0.00	0.06	0.03	0.03	0.01	0.01	0.07	0.02	0.06	0.06	0.07	0.11	0.14	0.10	0.13
Central Latin America	0.02	0.01	0.01	0.02	0.01	0.00	0.00	0.01	0.01	0.00	0.01	0.00	0.00	0.01	0.00	0.04	0.04	0.03	0.01	0.01	0.05	0.02	0.08	0.06	0.08	0.11	0.10	0.12	0.12
Southern Latin America	0.03	0.02	0.02	0.03	0.00	0.00	0.00	0.00	0.00	0.01	0.01	0.00	0.00	0.01	0.01	0.05	0.03	0.04	0.02	0.01	0.05	0.01	0.10	0.09	0.07	0.07	0.06	0.14	0.11
Tropical Latin America	0.01	0.02	0.01	0.01	0.00	0.01	0.01	0.01	0.01	0.01	0.01	0.02	0.00	0.01	0.02	0.04	0.03	0.03	0.04	0.02	0.08	0.01	0.09	0.07	0.08	0.07	0.07	0.13	0.10
North Africa and Middle East	0.01	0.01	0.01	0.00	0.01	0.00	0.00	0.00	0.01	0.02	0.01	0.00	0.01	0.01	0.01	0.05	0.02	0.03	0.02	0.01	0.07	0.05	0.08	0.10	0.09	0.10	0.08	0.16	0.03
High−income North America	0.02	0.04	0.00	0.01	0.00	0.00	0.00	0.00	0.01	0.01	0.01	0.00	0.00	0.01	0.00	0.04	0.03	0.05	0.02	0.01	0.08	0.03	0.12	0.13	0.07	0.06	0.03	0.14	0.04
Oceania	0.00	0.00	0.00	0.00	0.00	0.01	0.00	0.00	0.01	0.01	0.00	0.00	0.01	0.01	0.00	0.02	0.01	0.01	0.01	0.02	0.03	0.02	0.05	0.09	0.07	0.07	0.11	0.32	0.12
Central Sub−Saharan Africa	0.01	0.01	0.00	0.01	0.00	0.01	0.00	0.00	0.01	0.01	0.01	0.00	0.01	0.01	0.01	0.04	0.02	0.02	0.05	0.02	0.03	0.02	0.05	0.07	0.10	0.06	0.06	0.17	0.21
Eastern Sub−Saharan Africa	0.01	0.01	0.00	0.01	0.01	0.00	0.00	0.00	0.01	0.01	0.01	0.01	0.02	0.02	0.01	0.03	0.03	0.01	0.06	0.02	0.03	0.02	0.05	0.03	0.15	0.06	0.06	0.12	0.19
Southern Sub−Saharan Africa	0.01	0.01	0.00	0.00	0.00	0.01	0.00	0.00	0.01	0.01	0.01	0.01	0.01	0.01	0.01	0.07	0.02	0.03	0.06	0.02	0.03	0.08	0.06	0.08	0.06	0.05	0.04	0.13	0.17
Western Sub−Saharan Africa	0.01	0.01	0.00	0.00	0.00	0.01	0.00	0.00	0.00	0.01	0.00	0.00	0.01	0.03	0.01	0.05	0.02	0.02	0.02	0.01	0.04	0.05	0.05	0.05	0.08	0.06	0.06	0.19	0.19

0.3 0.25 0.2 0.15 0.1 0.05

A Figure S4

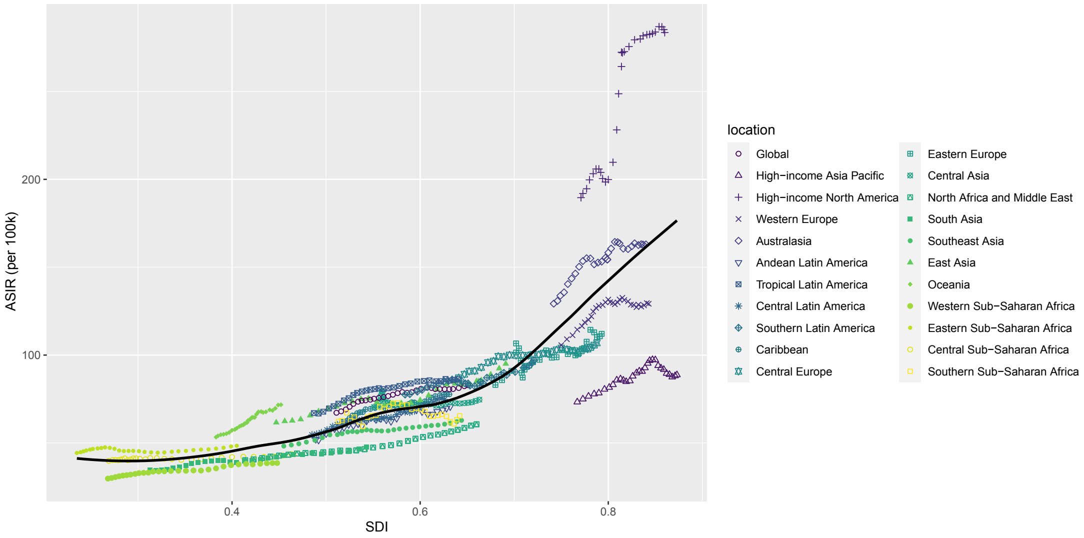

B

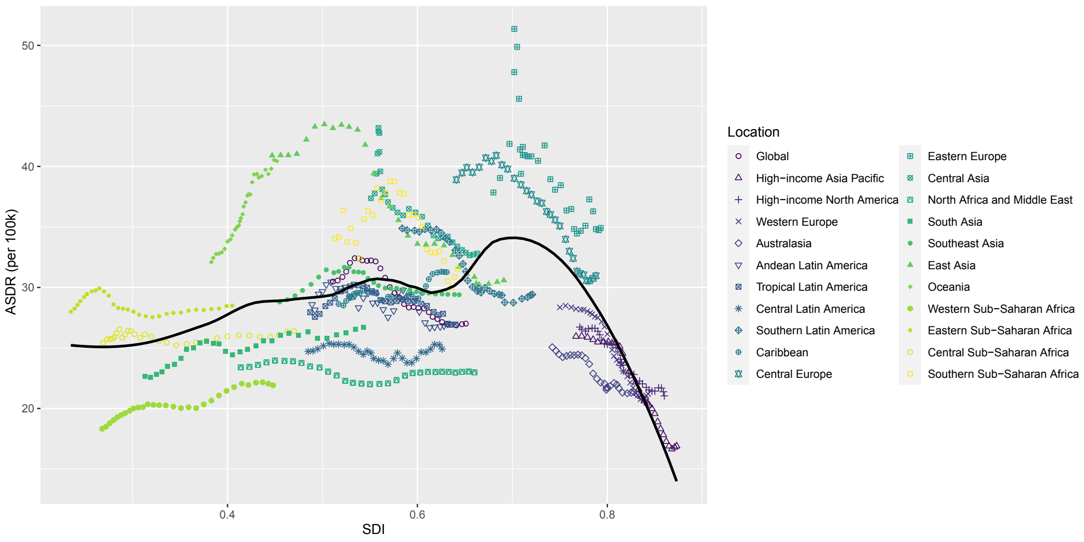

C

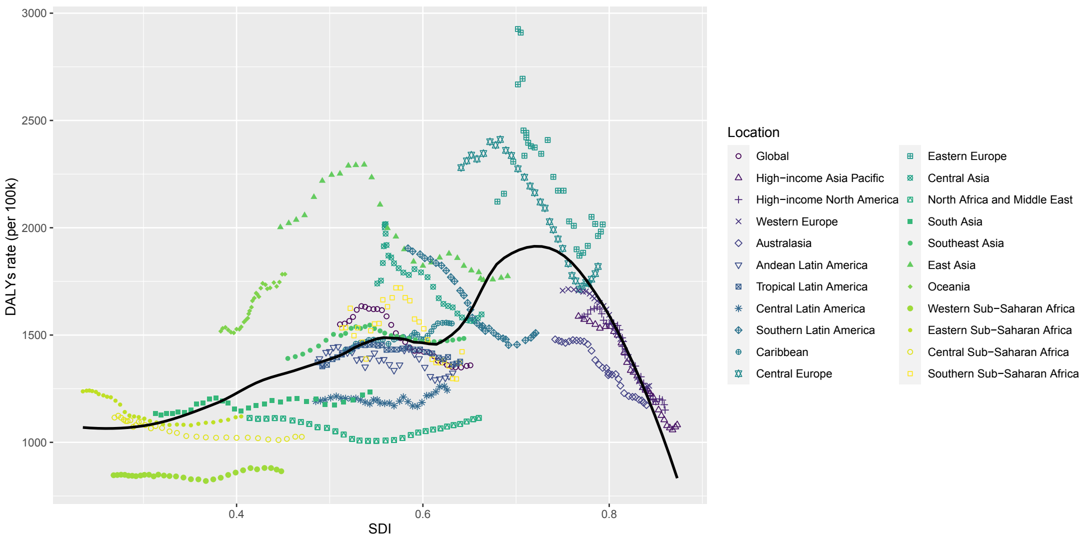

Figure S5

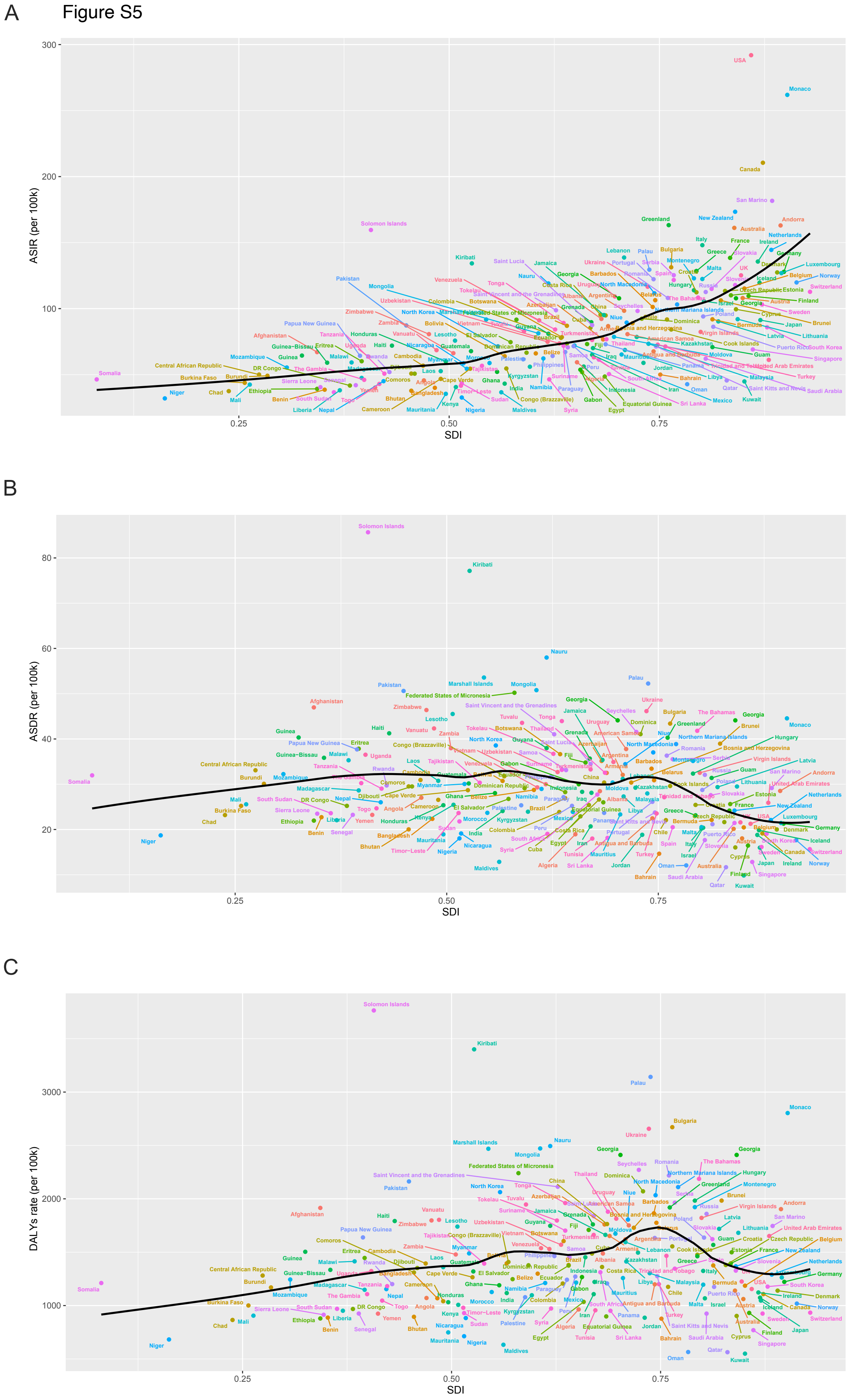
A
ASIR (per 100k)
300
200
100
0.25
0.50
0.75
SDI
B
ASDR (per 100k)
80
60
40
20
0.25
0.50
0.75
SDI
C
DALYs rate (per 100k)
3000
2000
1000
0.25
0.50
0.75
SDI

A

Figure S6

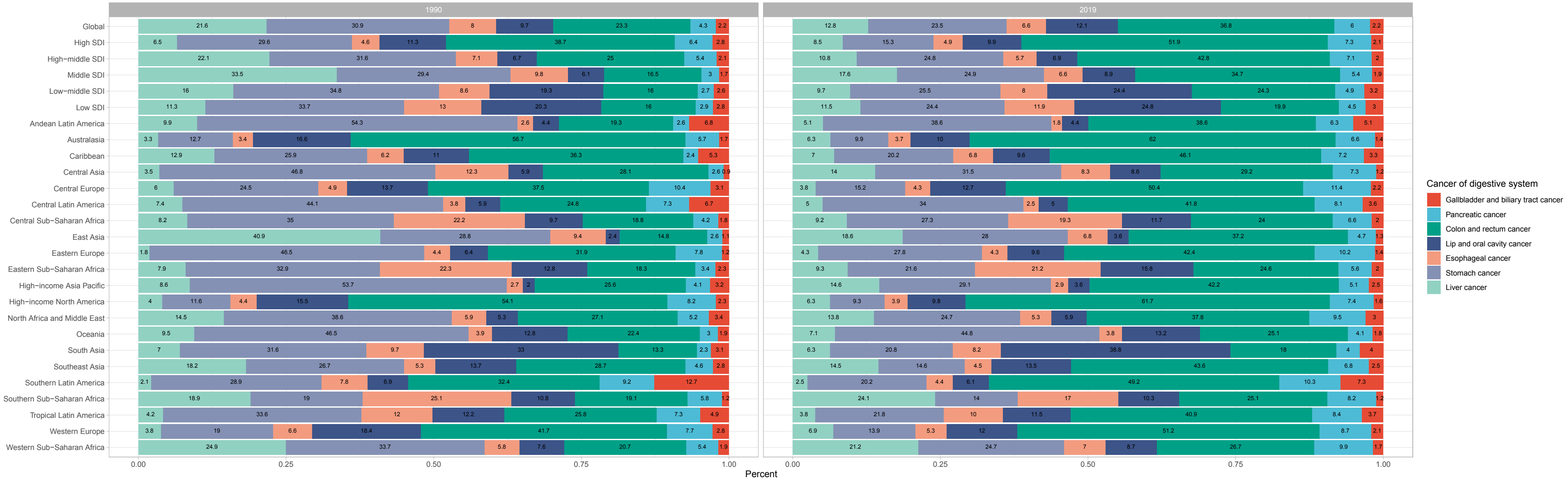
1990
2019
Global
High SDI
High−middle SDI
Middle SDI
Low−middle SDI
Low SDI
Andean Latin America
Australasia
Caribbean
Central Asia
Central Europe
Central Latin America
Central Sub−Saharan Africa
East Asia
Eastern Europe
Eastern Sub−Saharan Africa
High−income Asia Pacific
High−income North America
North Africa and Middle East
Oceania
South Asia
Southeast Asia
Southern Latin America
Southern Sub−Saharan Africa
Tropical Latin America
Western Europe
Western Sub−Saharan Africa
0.00
0.25
0.50
0.75
1.00
Percent
Cancer of digestive system
Gallbladder and biliary tract cancer
Pancreatic cancer
Colon and rectum cancer
Lip and oral cavity cancer
Esophageal cancer
Stomach cancer
Liver cancer

B

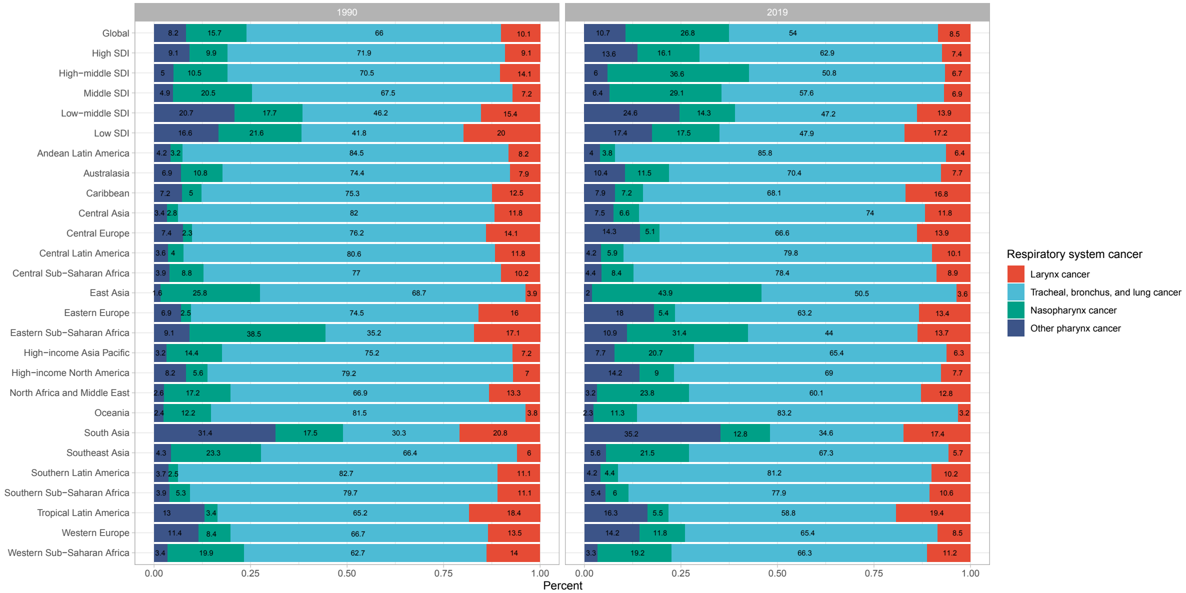
1990
2019
Global
High SDI
High−middle SDI
Middle SDI
Low−middle SDI
Low SDI
Andean Latin America
Australasia
Caribbean
Central Asia
Central Europe
Central Latin America
Central Sub−Saharan Africa
East Asia
Eastern Europe
Eastern Sub−Saharan Africa
High−income Asia Pacific
High−income North America
North Africa and Middle East
Oceania
South Asia
Southeast Asia
Southern Latin America
Southern Sub−Saharan Africa
Tropical Latin America
Western Europe
Western Sub−Saharan Africa
0.00
0.25
0.50
0.75
1.00
Percent
Respiratory system cancer
Larynx cancer
Tracheal, bronchus, and lung cancer
Nasopharynx cancer
Other pharynx cancer

Figure S7

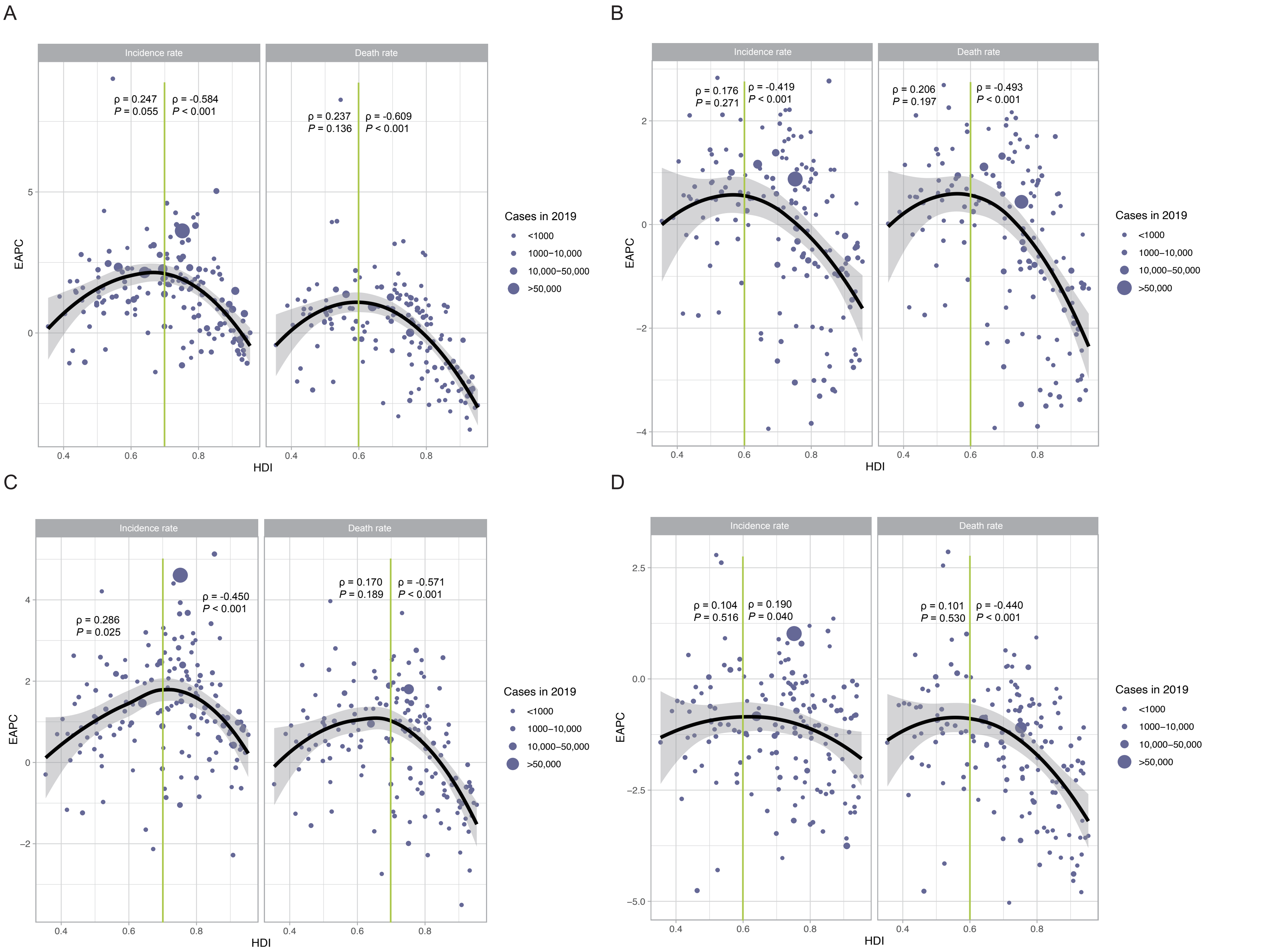
A
Incidence rate
Death rate
ρ = 0.247
P = 0.055
ρ = -0.584
P < 0.001
ρ = 0.237
P = 0.136
ρ = -0.609
P < 0.001
EAPC
HDI
Cases in 2019
<1000
1000–10,000
10,000–50,000
>50,000
B
Incidence rate
Death rate
ρ = 0.176
P = 0.271
ρ = -0.419
P < 0.001
ρ = 0.206
P = 0.197
ρ = -0.493
P < 0.001
C
Incidence rate
Death rate
ρ = 0.286
P = 0.025
ρ = -0.450
P < 0.001
ρ = 0.170
P = 0.189
ρ = -0.571
P < 0.001
D
Incidence rate
Death rate
ρ = 0.104
P = 0.516
ρ = 0.190
P = 0.040
ρ = 0.101
P = 0.530
ρ = -0.440
P < 0.001

Figure S8

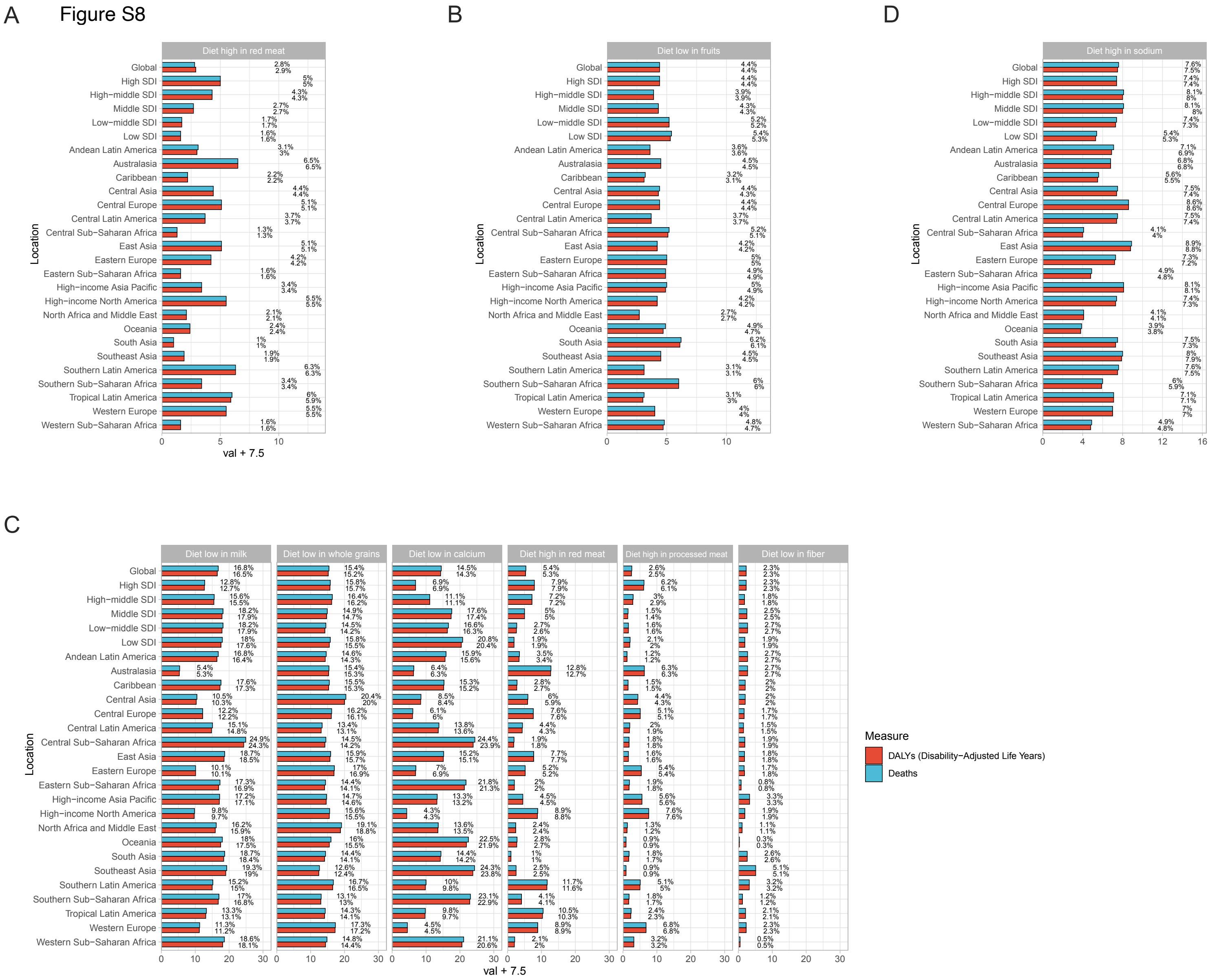
A
B
D
C
Diet high in red meat
Diet low in fruits
Diet high in sodium
Diet low in milk
Diet low in whole grains
Diet low in calcium
Diet high in red meat
Diet high in processed meat
Diet low in fiber
Location
val + 7.5
Global
High SDI
High−middle SDI
Middle SDI
Low−middle SDI
Low SDI
Andean Latin America
Australasia
Caribbean
Central Asia
Central Europe
Central Latin America
Central Sub−Saharan Africa
East Asia
Eastern Europe
Eastern Sub−Saharan Africa
High−income Asia Pacific
High−income North America
North Africa and Middle East
Oceania
South Asia
Southeast Asia
Southern Latin America
Southern Sub−Saharan Africa
Tropical Latin America
Western Europe
Western Sub−Saharan Africa
Measure
DALYs (Disability−Adjusted Life Years)
Deaths

Figure S9

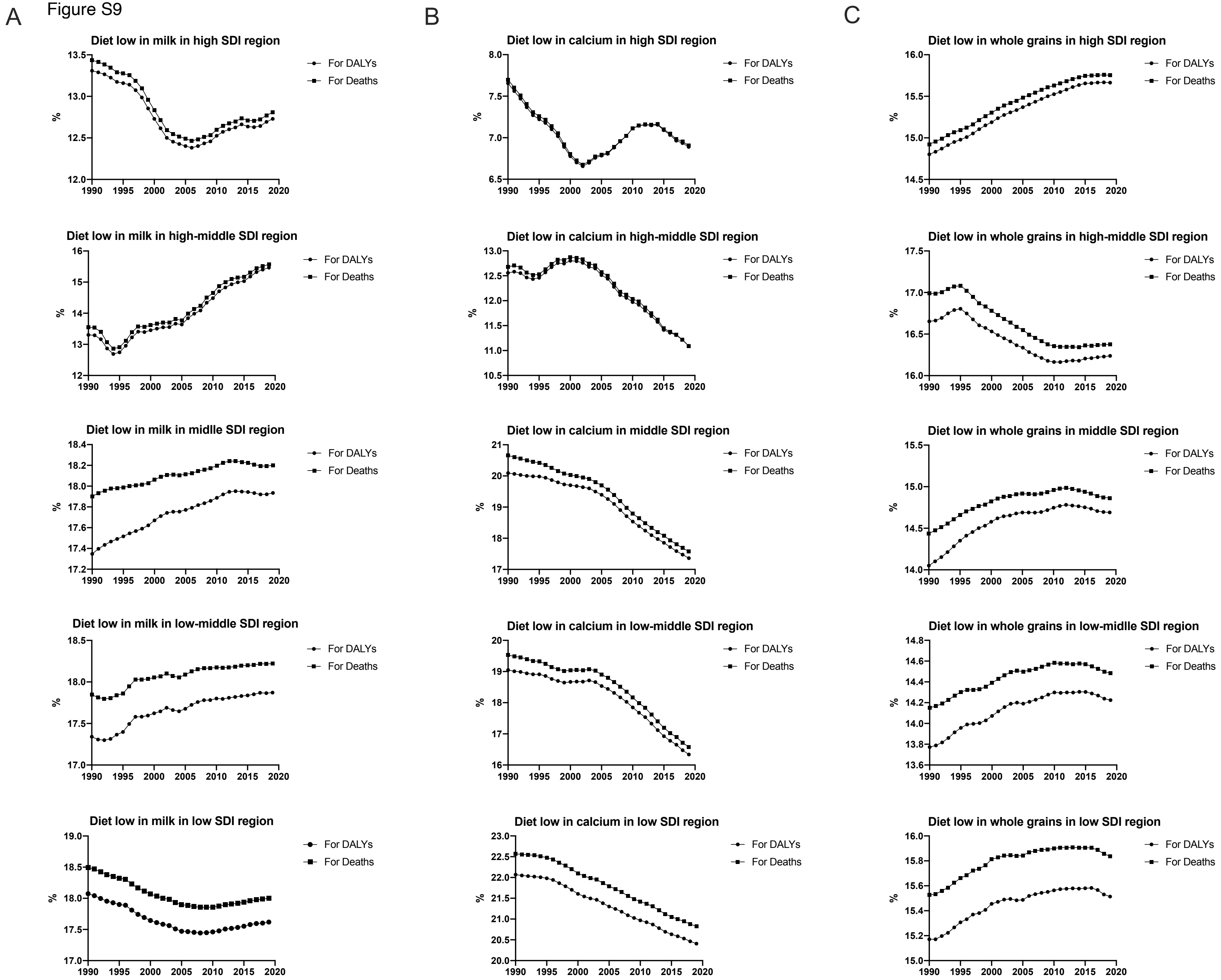
A
B
C
Diet low in milk in high SDI region
Diet low in calcium in high SDI region
Diet low in whole grains in high SDI region
Diet low in milk in high-middle SDI region
Diet low in calcium in high-middle SDI region
Diet low in whole grains in high-middle SDI region
Diet low in milk in midlle SDI region
Diet low in calcium in middle SDI region
Diet low in whole grains in middle SDI region
Diet low in milk in low-middle SDI region
Diet low in calcium in low-middle SDI region
Diet low in whole grains in low-midlle SDI region
Diet low in milk in low SDI region
Diet low in calcium in low SDI region
Diet low in whole grains in low SDI region
For DALYs
For Deaths
%

Figure S10

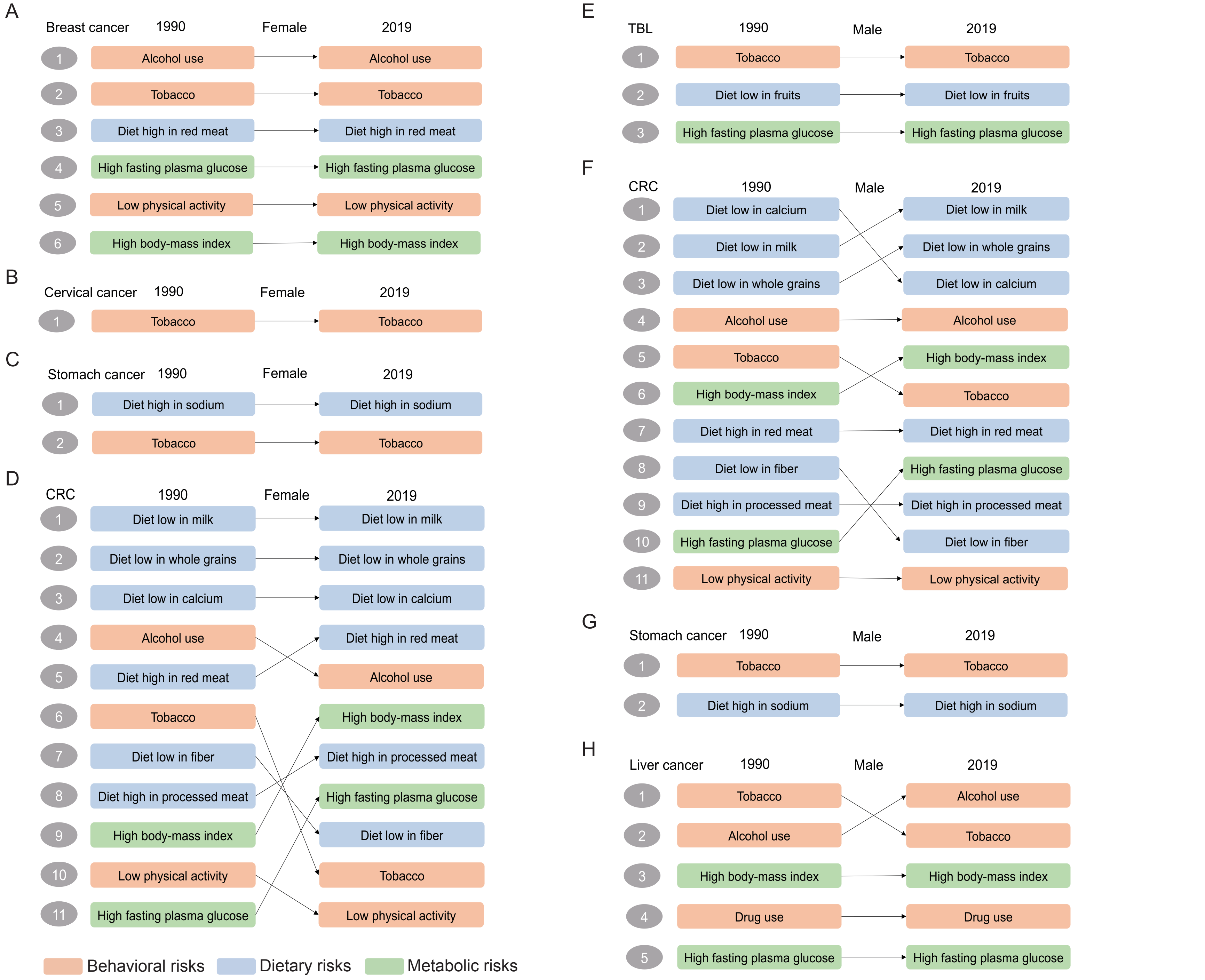
A
Breast cancer 1990 Female 2019
1 Alcohol use → Alcohol use
2 Tobacco → Tobacco
3 Diet high in red meat → Diet high in red meat
4 High fasting plasma glucose → High fasting plasma glucose
5 Low physical activity → Low physical activity
6 High body-mass index → High body-mass index
B
Cervical cancer 1990 Female 2019
1 Tobacco → Tobacco
C
Stomach cancer 1990 Female 2019
1 Diet high in sodium → Diet high in sodium
2 Tobacco → Tobacco
D
CRC 1990 Female 2019
1 Diet low in milk / Diet low in milk
2 Diet low in whole grains / Diet low in whole grains
3 Diet low in calcium / Diet low in calcium
4 Alcohol use / Diet high in red meat
5 Diet high in red meat / Alcohol use
6 Tobacco / High body-mass index
7 Diet low in fiber / Diet high in processed meat
8 Diet high in processed meat / High fasting plasma glucose
9 High body-mass index / Diet low in fiber
10 Low physical activity / Tobacco
11 High fasting plasma glucose / Low physical activity
E
TBL 1990 Male 2019
1 Tobacco → Tobacco
2 Diet low in fruits → Diet low in fruits
3 High fasting plasma glucose → High fasting plasma glucose
F
CRC 1990 Male 2019
1 Diet low in calcium / Diet low in milk
2 Diet low in milk / Diet low in whole grains
3 Diet low in whole grains / Diet low in calcium
4 Alcohol use / Alcohol use
5 Tobacco / High body-mass index
6 High body-mass index / Tobacco
7 Diet high in red meat / Diet high in red meat
8 Diet low in fiber / High fasting plasma glucose
9 Diet high in processed meat / Diet high in processed meat
10 High fasting plasma glucose / Diet low in fiber
11 Low physical activity / Low physical activity
G
Stomach cancer 1990 Male 2019
1 Tobacco → Tobacco
2 Diet high in sodium → Diet high in sodium
H
Liver cancer 1990 Male 2019
1 Tobacco / Alcohol use
2 Alcohol use / Tobacco
3 High body-mass index / High body-mass index
4 Drug use / Drug use
5 High fasting plasma glucose / High fasting plasma glucose
Behavioral risks
Dietary risks
Metabolic risks

BMJ Publishing Group Limited (BMJ) disclaims all liability and responsibility arising from any reliance
placed on this supplemental material which has been supplied by the author(s)

Figure S11

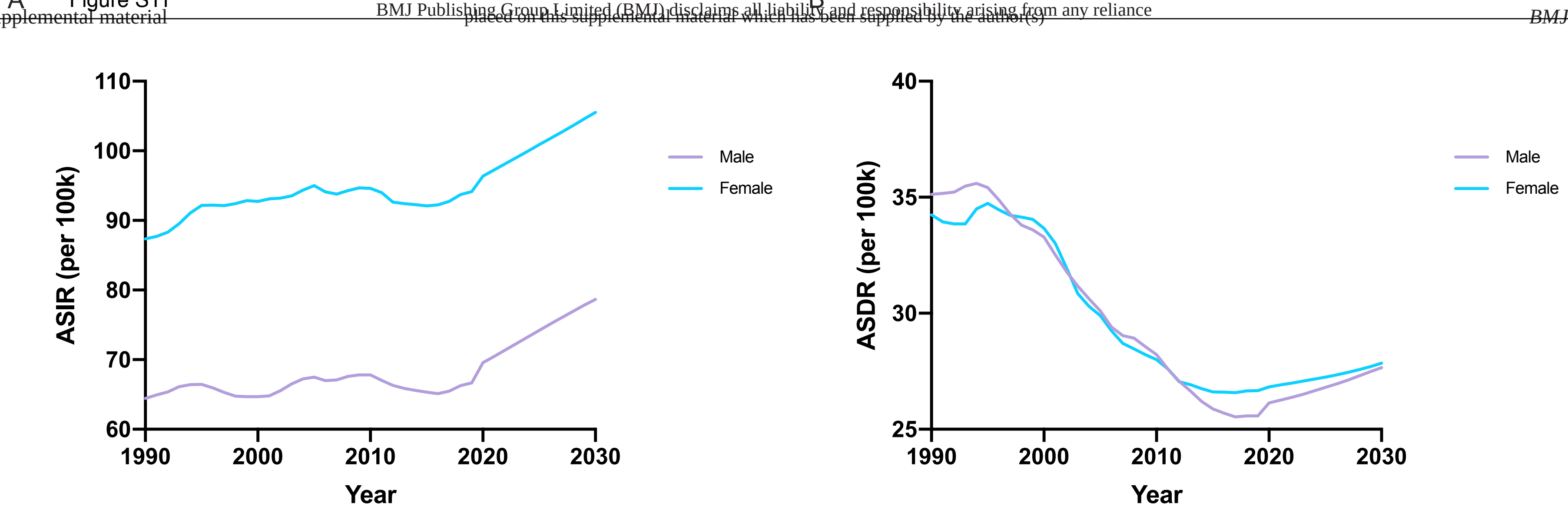

Supplement: Supplementary data [file bmjonc-2023-000049supp001.pdf]
